# Targeting the *PBX1–BCL2L1* axis as a therapeutic strategy in colorectal cancer

**DOI:** 10.1038/s41420-026-03139-2

**Published:** 2026-05-05

**Authors:** Hao Lin, Ting Su, Ying Liu, Ruilan Deng, Jie Li, Xuanhao Lin, Qiaoling Ke, Yijing Luo, Lele Meng, Bin Liang, Xuhong Song, Dongyang Huang, Lingzhu Xie

**Affiliations:** 1 Department of Gastroenterology, Shantou Central Hospital, Shantou, China; 2 Department of Cell Biology and Genetics, Key Laboratory of Molecular Biology in High Cancer Incidence Coastal Chaoshan Area of Guangdong Higher Education Institutes, Shantou University Medical College, Shantou, China; 3 Department of Biobank, Shantou Central Hospital, Shantou, China; 4 Department of Gastroenterology, First Affiliated Hospital of Shantou University Medical College, Shantou, China

**Keywords:** Mechanisms of disease, Colorectal cancer

## Abstract

*Pre-B-cell leukemia homeobox 1* (*PBX1*) is a transcription factor involved in diverse cellular processes, but its role in colorectal cancer (CRC) remains incompletely understood. In this study, we show that *PBX1* is downregulated in CRC tissues and cell lines. Functional experiments revealed that *PBX1* overexpression inhibits proliferation, migration, and invasion, but paradoxically suppresses apoptosis, suggesting a dual regulatory role. Transcriptome and CUT&Tag profiling identified *BCL2L1* as a direct transcriptional target of PBX1. PBX1 binds the *BCL2L1* promoter and enhances Bcl-xL expression, contributing to apoptotic resistance. *BCL2L1* knockdown reversed the anti-apoptotic effects of *PBX1* and restored apoptosis levels. Upon 5-fluorouracil (5-FU) treatment, *PBX1* overexpression reduced cell viability, while concurrent *BCL2L1* knockdown significantly enhanced drug sensitivity. In vivo, xenograft experiments demonstrated that *PBX1* overexpression suppressed tumor growth, which was further augmented by *BCL2L1* knockdown. These results support the dual role of *PBX1* in simultaneously inhibiting tumor growth while promoting cell survival through the *BCL2L1*–Bcl-xL axis. This regulatory interaction may influence tumor persistence and therapeutic response in CRC.

## Introduction

Colorectal cancer (CRC) is the third most common cancer worldwide, representing 10.0% of all cancer cases, and the second leading cause of cancer-related deaths, contributing to 9.4% of total cancer deaths in 2020 [1]. Despite advancements in surgical techniques, chemotherapy, and targeted therapies, the recurrence and mortality rates for CRC remain high [2]. Tumor recurrence, driven by the complex interplay of molecular pathways regulating cell proliferation, survival, and apoptosis, continues to challenge effective treatment. Understanding the key molecular regulators involved in these processes is crucial for developing more effective therapies and improving patient outcomes.

Among the transcription factors implicated in cancer biology, the *Pre-B-cell leukemia homeobox 1* (*PBX1*) gene has garnered attention for its dual role in various cancers [3–6]. *PBX1*, a member of the TALE (three amino acid loop extension) homeodomain family, is recognized for its role in developmental processes and gene expression regulation [7–9]. Depending on the cellular and molecular context, *PBX1* can act as either a tumor suppressor or an oncogene in different types of cancer. For instance, in pre-B cell acute lymphoblastic leukemia, *PBX1* was first identified as an oncogene through the t(1;19) chromosomal translocation, which produces the E2A–PBX1 fusion protein that drives leukemogenesis [10]. Similarly, *PBX1* has been shown to promote tumor progression, chemoresistance, and stemness in breast, ovarian, and clear cell renal carcinomas [11, 12]. However, in CRC, *PBX1* appears to play a distinct role. While *PBX1* expression is frequently downregulated in CRC tissues, forced overexpression of *PBX1* has been shown to suppress tumor cell proliferation, migration, and invasion, suggesting a tumor-suppressive function [13]. However, the precise mechanisms by which *PBX1* influences CRC cell fate remain poorly understood.

In addition to its role in cell proliferation, *PBX1* has been implicated in regulating apoptosis across various cancers. In lung cancer, *PBX1* silencing induces apoptosis through ROS production and inhibition of the *STAT3—Bcl-2*–Survivin pathway [14]. In breast cancer, *PBX1* is negatively regulated by the lncRNA uc.38, which induces apoptosis by downregulating Bcl-2 family proteins [15]. Moreover, in prostate cancer, *PBX1*’s stability, regulated by the deubiquitinase USP9x, plays a critical role in resistance to apoptosis, positioning *PBX1* as a potential therapeutic target to overcome chemoresistance [16]. These studies underscore *PBX1*’s critical role in the regulation of apoptosis and suggests that similar mechanisms may exist in other cancers.

Apoptosis, a crucial mechanism for maintaining cellular homeostasis and preventing tumor growth, is often disrupted in cancer, leading to tumor recurrence and resistance to treatment. The Bcl-2 family of proteins, particularly BCL2L1, plays a pivotal role in regulating apoptosis. BCL2L1 encodes two isoforms: Bcl-xS, which promotes apoptosis, and Bcl-xL, which inhibits apoptosis and supports cell survival [17–19]. These isoforms are generated by alternative splicing, resulting in structural differences that influence their roles in apoptosis regulation [20]. Bcl-xL, with its intact BH1, BH2, and C-terminal hydrophobic domain, prevents apoptosis by binding to and inhibiting pro-apoptotic proteins such as Bax and Bak [21]. In contrast, Bcl-xS, lacking key anti-apoptotic domains, allows apoptotic pathways to proceed [22]. While several upstream signaling pathways and splice factors have been shown to modulate the balance between Bcl-xL and Bcl-xS [17, 23], the transcriptional regulation of *BCL2L1*, particularly in CRC, remains poorly defined.

This gap in understanding is crucial, as apoptosis and proliferation are closely linked in tumor progression. Our study investigates how *PBX1* influences cell both cell proliferation and apoptosis, focusing on its regulation of *BCL2L1*. By defining the *PBX1*–*BCL2L1* axis, this research may provide new therapeutic insights into strategies that simultaneously suppress tumor growth and enhance apoptosis sensitivity in CRC.

## Result

### Downregulation of *PBX1* and subtype-specific heterogeneity in CRC

**Fig. 1:**
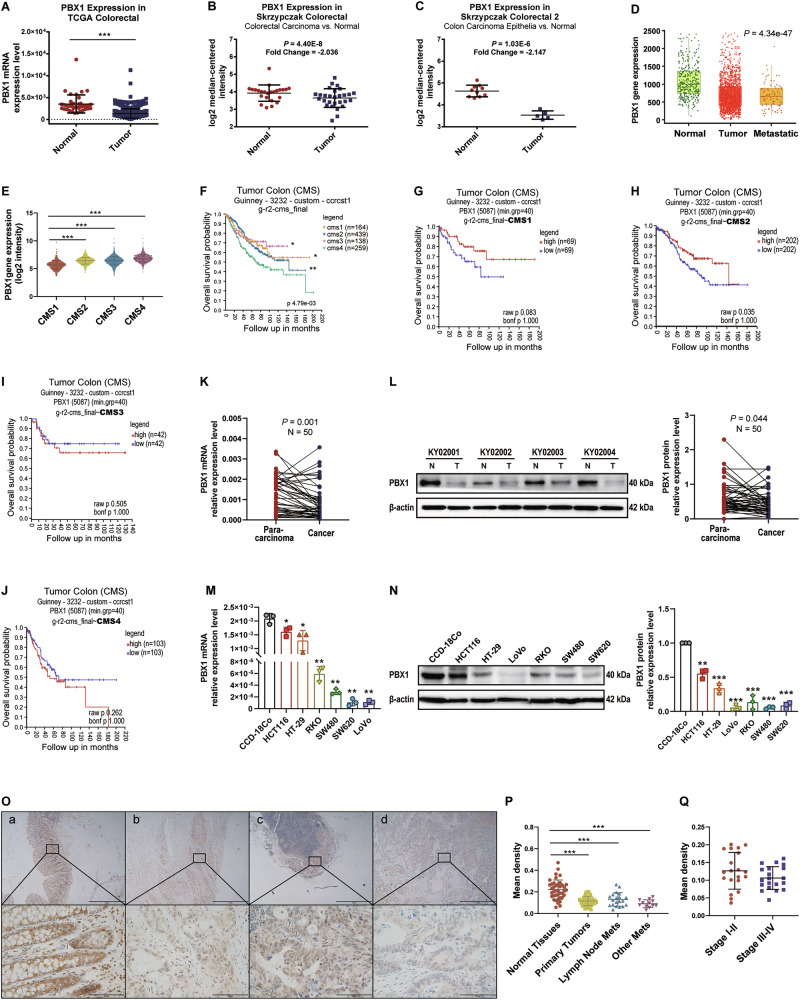
*PBX1* is downregulated in colorectal cancer and shows CMS-dependent prognostic relevance. **A** *PBX1* mRNA expression levels in CRC vs. normal tissues analyzed from TCGA RNA-seq data. **B**, **C** *PBX1* expression in Oncomine datasets ‘Skrzypczak Colorectal’ and ‘Skrzypczak Colorectal 2’. **D** Box plot of *PBX1* mRNA expression levels in normal colon (N), primary colon tumor (T), and metastatic (M) tissues. The analysis was performed using the TNMplot online tool (https://www.tnmplot.com), based on data from the TCGA and GTEx cohorts. The global Kruskal-Wallis p-value is shown. **E** *PBX1* expression across CMS subtypes (CMS1–4) using the R2 Genomics Analysis and Visualization Platform. **F** Kaplan–Meier survival analysis of *PBX1* expression in CRC subtypes (CMS1-4). CMS4 exhibits significantly lower survival compared to CMS1, CMS2, and CMS3 (* or ** indicate comparisons with CMS4; log-rank test). Kaplan-Meier survival analysis of *PBX1* expression in CMS1 (**G**), CMS2 (**H**), CMS3 (**I**), and CMS4 (**J**) using the log-rank test. **K**, **L** qPCR and western blot showing lower *PBX1* expression in CRC tumor tissues vs. adjacent normal tissues from 50 patients. **M**, **N** *PBX1* expression in normal colon cells and CRC cell lines. **O** Immunohistochemical (IHC) staining showing *PBX1* expression in normal colon tissue (panel a), primary tumor (panel b), lymph node metastasis (panel c), and liver metastatic tissues (panel d). **P** Statistical analysis of the mean density of *PBX1* staining, with significant differences observed between normal tissues and primary tumors, lymph node metastasis, and other metastatic tissues. **Q** Comparison of *PBX1* expression in primary tumors across different TNM stages, showing no significant difference in *PBX1* levels between Stage I-II and Stage III-IV tumors (*p* = 0. 142). Data are shown as mean ± S.D., *n* >= 3. *P < 0.05, **P < 0.01, ***P < 0.001.

Analysis of both TCGA and Oncomine databases revealed a significant reduction in *PBX1* mRNA expression in CRC tissues compared to normal tissues, suggesting a potential tumor-suppressive role for *PBX1* in CRC. Specifically, TCGA RNA-Seq data [24] showed that *PBX1* expression was significantly lower in CRC tissues than in adjacent normal tissues (*P* < 0.001) (Fig. 1A). Consistent with this, analysis of the “Skrzypczak Colorectal” and “Skrzypczak Colorectal 2” datasets [25], from Oncomine further confirmed *PBX1* downregulation in CRC tumor tissues, with fold changes of -2.036 and -2.147, and P-values of 4.40 × 10^-8^ and 1.03 × 10^-6^, respectively (Fig. 1B, C).

Further analysis using the TNMplot database demonstrated that *PBX1* expression was significantly reduced in both primary (*P* = 5.60 × 10⁻⁴⁸, FC = 0.59) and metastatic tumors (*P* = 3.14 × 10⁻¹⁴) compared with normal mucosa, while no significant difference was observed between the two tumor groups (*P* = 0.467) (Fig. 1D). Notably, the upper quartile (Q3) and maximum expression values progressively decreased from normal tissue to metastasis (Normal Q3 = 1461, Max = 7786; Tumor Q3 = 954, Max = 6070; Metastatic Q3 = 882.5, Max = 4722), suggesting a potential selection against *PBX1*-high clones during tumor progression (Supplementary Table S1).

To assess *PBX1* expression and its prognostic relevance across CRC subtypes, we analyzed the R2 Genomics Analysis and Visualization Platform based on the CMS classification. LSD multiple comparison analysis revealed significant differences in *PBX1* expression across CMS subtypes (*P* < 0.001), with *PBX1* expression lowest in CMS1 (immune subtype) (Fig. 1E). Kaplan-Meier survival analysis showed that CMS4 had significantly lower survival compared to CMS1, CMS2, and CMS3, with statistically significant differences observed between CMS4 and each of the other subtypes (*P* = 0.010, *P* = 0.002, *P* = 0.017, respectively) (Fig. 1F). Further survival analysis revealed subtype-specific associations between *PBX1* expression and patient outcomes (Fig. 1G-J): In CMS1, patients with low *PBX1* expression tended to have poorer overall survival, although the difference did not reach statistical significance (*P* = 0.083), suggesting a potential link between *PBX1* downregulation and immune-related tumor progression. In CMS2, high *PBX1* expression was significantly associated with better overall survival (*P* = 0.035), indicating that *PBX1* may help maintain epithelial differentiation and suppress malignant proliferation. In contrast, CMS3 and CMS4 showed no significant survival correlation with *PBX1* expression (*P* = 0.535 and *P* = 0.262, respectively).

Together, these bioinformatic results indicate that *PBX1* is generally down regulated in CRC and exhibits CMS subtype-dependent prognostic significance. Its low expression in CMS1 may be linked to immune evasion and poorer survival, while high expression in CMS2 correlates with better survival outcomes, suggesting *PBX1* as a tumor suppressor in this subtype.

To validate the bioinformatic observations, we analyzed tumor and adjacent non-tumor tissues from 50 CRC patients. qPCR and Western blot analyses confirmed that *PBX1* expression was significantly lower in tumor tissues compared to adjacent normal tissues (Fig. 1K, L), reinforcing the hypothesis that *PBX1* may act as a tumor suppressor in CRC. Immunohistochemical staining further corroborated these findings, revealing a marked reduction in PBX1 expression in primary tumors and metastatic tissues compared to normal tissues. As shown in Fig. 1O, PBX1 staining was prominent in normal colon tissue (panel a), with weaker staining observed in primary tumor samples (panel b) and lymph node metastases (panel c). The lowest expression was seen in distant metastatic tissues (panel d). Statistical analysis of the mean density of PBX1 staining across these samples confirmed significant differences, with primary tumors, lymph node metastases, and other metastatic tissues showing significantly lower expression compared to normal tissues (Fig. 1P, *p* < 0.001). Next, we compared PBX1 expression in primary tumors across different TNM stages. Figure 1Q shows that while the mean density of PBX1 in primary tumors from Stage I-II and Stage III-IV patients was lower than in normal tissues, there was no statistically significant difference between the two stages (Stage I-II vs. Stage III-IV). This suggests that PBX1 downregulation may be an early event in CRC development rather than being stage-dependent. To further substantiate this observation at the cellular level, we examined *PBX1* expression in multiple CRC cell lines (HCT116, HT-29, LoVo, RKO, SW480, SW620) and the normal colon cell line CCD-18Co. The results demonstrated that *PBX1* expression was significantly downregulated at both the mRNA and protein levels in CRC cell lines (Fig. 1M, N), providing additional support for the idea that *PBX1* downregulation is a consistent feature of CRC, occurring across both patient tissues and in vitro cellular models.

### *PBX1* generally functions as a tumor suppressor in CRC

**Fig. 2:**
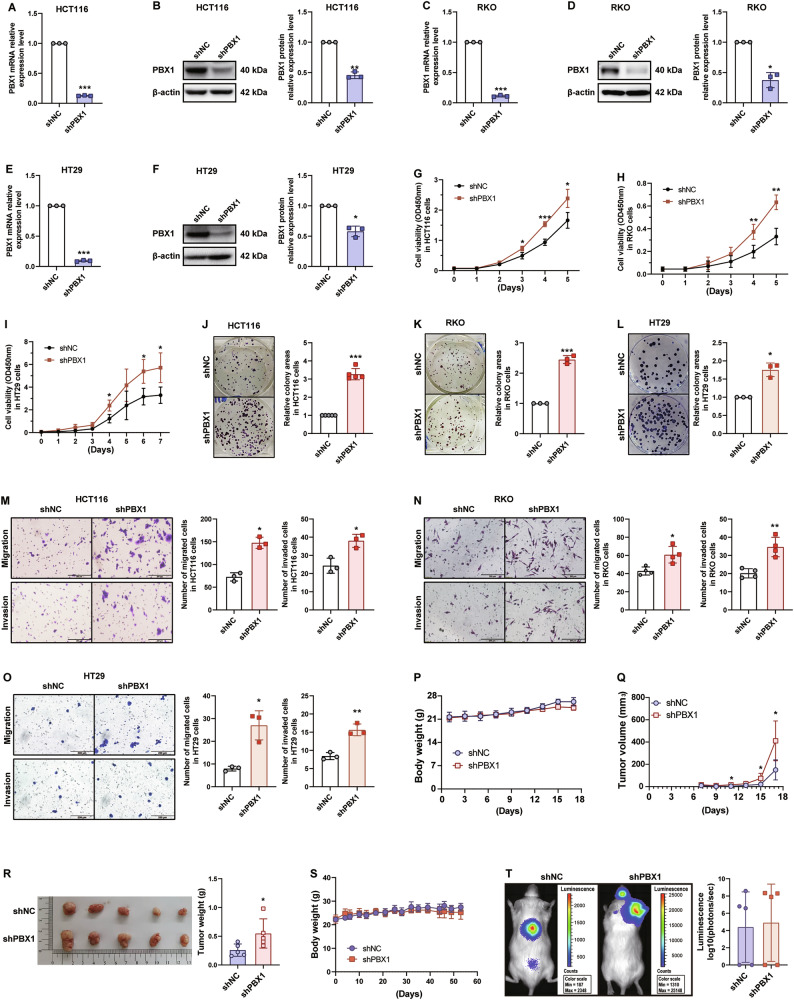
*PBX1* knockdown promotes malignant phenotypes in CRC cells. **A**–**F** Confirmation of *PBX1* knockdown in HCT116, RKO, and HT29 cells by qPCR and western blot. Functional assays show that *PBX1* knockdown significantly enhances cell proliferation (**G**–**I**), colony formation (**J**–**L**), migration and invasion (**M**–**O**) in HCT116, RKO, and HT29 cells. **P** Body weight measurements of nude mice injected with HCT116 cells with *PBX1* knockdown (shPBX1) or control (shNC), showing no significant difference between groups. **Q** Tumor growth curve showing that subcutaneous tumors in the shPBX1 group are significantly larger than those in the shNC group at later stages of the experiment. **R** Weight of subcutaneous tumors, showing that tumors from the shPBX1 group are significantly heavier than those from the shNC group. **S**, **T** Assessment of metastatic potential in NOD-SCID mice injected with HCT116 cells via tail vein, with no significant differences in body weight or metastatic spread between the shPBX1 and shNC groups. Significance for all data was determined by the independent samples t-test. Data are shown as mean ± S.D., *n* >= 3. *P < 0.05, **P < 0.01, ***P < 0.001.

**Fig. 3:**
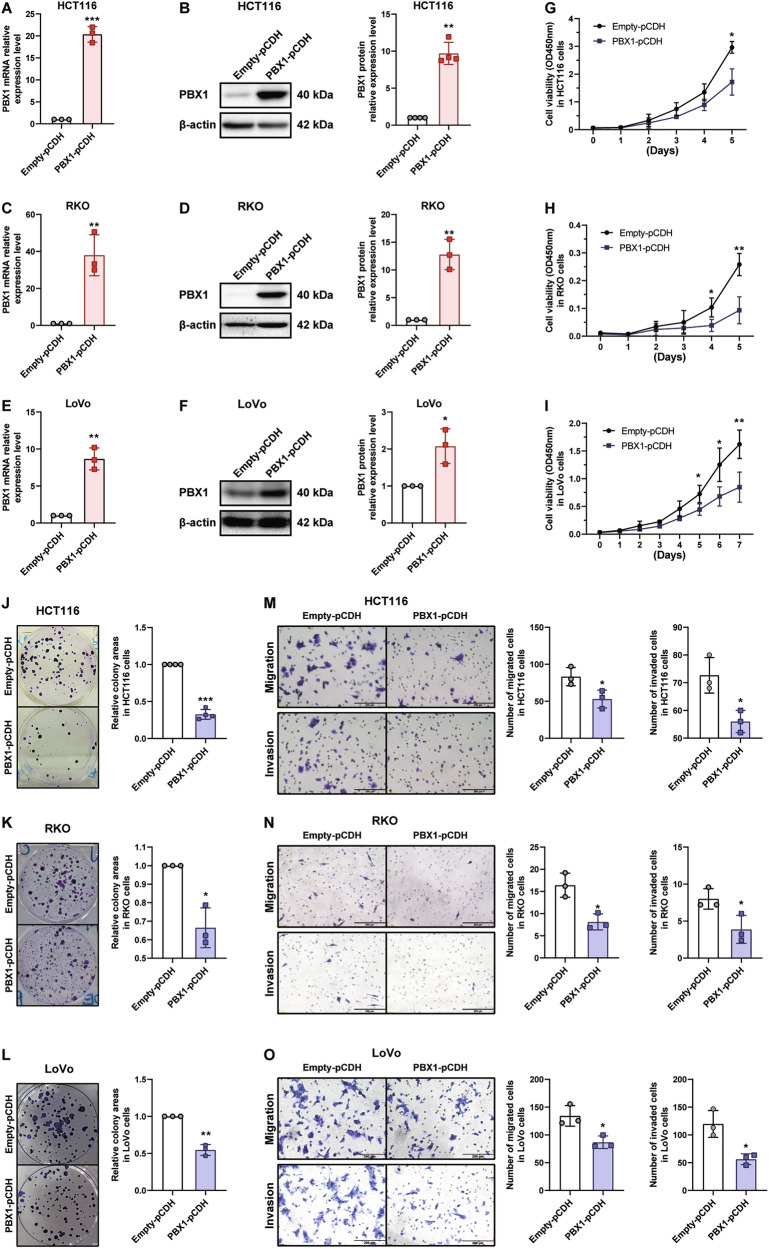
*PBX1* overexpression suppresses CRC progression. **A**–**F** Confirmation of *PBX1* overexpression by qPCR and western blot in HCT116, RKO, and LoVo cells. Functional assays indicate reduced proliferation **G**–**I**, colony formation (**J**–**L**), migration and invasion (**M**–**O**) upon *PBX1* overexpression in HCT116, RKO, and LoVo cells. Significance for all data was determined by the independent samples t-test. Data are shown as mean ± S.D., n >= 3. *P < 0.05, **P < 0.01, ***P < 0.001.

Building on the observed downregulation of *PBX1* in CRC tissues and cell lines, we further investigated its functional role in CRC progression by modulating its expression in vitro. Knockdown of *PBX1* in HCT116, RKO, and HT29 cells using shRNA significantly decreased *PBX1* mRNA and protein levels (Fig. 2A-F), which led to a significant increase in cell proliferation, colony formation, migration, and invasion (Fig. 2G-O). Conversely, overexpression of *PBX1* in HCT116, RKO, and LoVo cells using a lentiviral vector significantly increased *PBX1* mRNA and protein levels (Fig. 3A-F), which led to a significant suppression in cell proliferation, migration, invasion, and colony formation (Fig. 3G-O). These results suggest that *PBX1* generally functions as a tumor suppressor in CRC.

To further investigate the role of *PBX1* in vivo, we performed subcutaneous xenograft experiments. HCT116 with *PBX1* knockdown (shPBX1) or control (shNC) was injected subcutaneously into the flanks of nude mice. No significant differences in body weight were observed between the two groups (Fig. 2P). However, in the later stages of the experiment, tumors in the shPBX1 group were significantly larger than those in the shNC group (Fig. 2Q). Furthermore, the weight of subcutaneous tumors in the shPBX1 group was significantly greater than that in the shNC group (Fig. 2R).

Additionally, the same cell lines and groups were injected via tail vein into NOD-SCID mice to assess metastatic potential. No significant differences in body weight or metastatic spread were observed between the two groups (Fig. 2S, T).

### *BCL2L1* as a potential direct target of PBX1

**Fig. 4:**
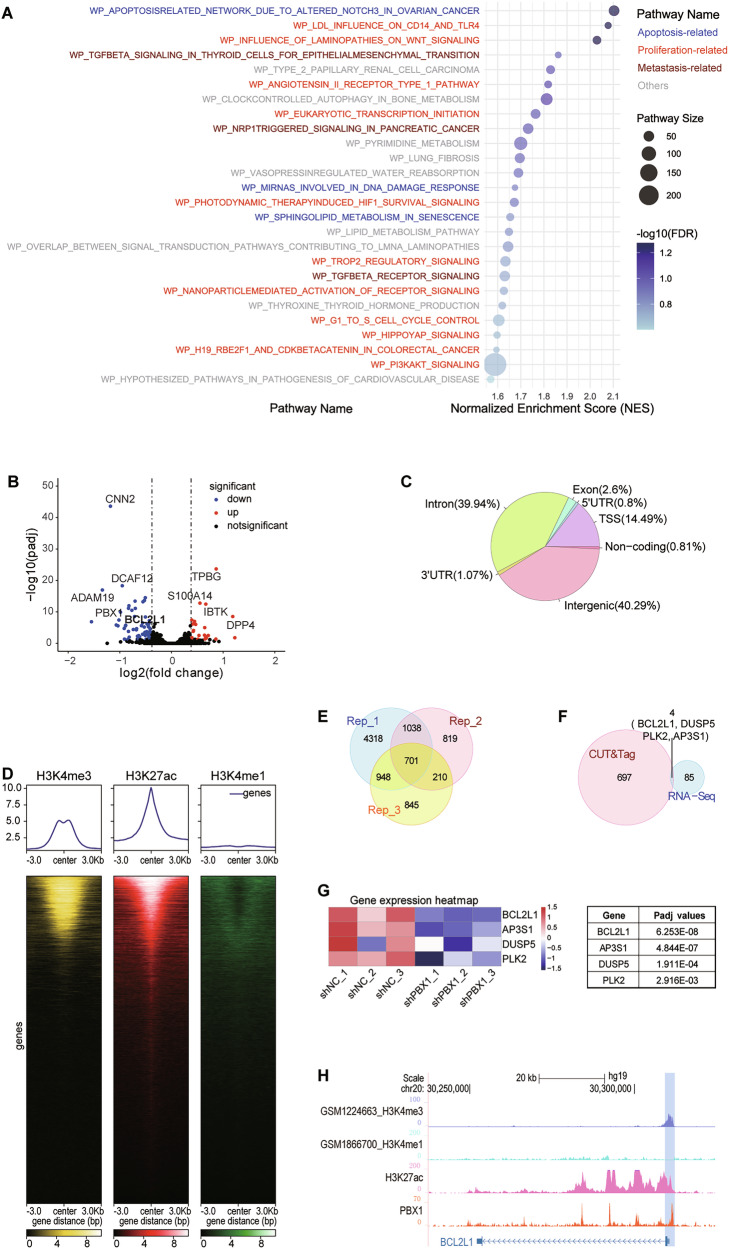
Integrated transcriptomic and epigenomic profiling identifies *BCL2L1* as a potential *PBX1* target. **A** GSEA shows enrichment of apoptosis- and proliferation-related pathways after *PBX1* knockdown. **B** Volcano plot of differentially expressed genes following *PBX1* knockdown. **C** Genomic distribution of *PBX1* binding sites identified by CUT&Tag. **D** Histone modification profiles at *PBX1*-bound regions show promoter-associated chromatin marks. **E** Venn diagram indicating 701 genes with PBX1 promoter binding. **F** Overlap between CUT&Tag and RNA-seq reveals four direct PBX1 targets. **G** Heatmap showing the expression levels of four direct PBX1 target genes; *BCL2L1* shows the lowest adjusted p-value. **H** CUT&Tag profile shows PBX1 binding at *BCL2L1* promoter.

To investigate the downstream genes and pathways regulated by PBX1, we performed RNA sequencing (RNA-seq) analysis in HCT116 cells following *PBX1* knockdown. The pathways significantly affected are summarized in Fig. 4A, while representative differentially expressed genes are shown in Fig. 4B. As Fig. 4A shown, *PBX1* knockdown in HCT116 cells led to significant enrichment of gene sets related to apoptosis, cell proliferation, and metastasis. The top-ranked pathway was the “Apoptosis-Related Network due to Altered Notch3” (NES = 2.10, FDR = 0.053), suggesting that reduced *PBX1* expression may enhance pro-apoptotic signaling. Additional pathways enriched in the knockdown condition included “LDL Influence on CD14 and TLR4” and “Influence of Laminopathies on Wnt Signaling”, both associated with immune regulation and proliferative activity [26], as well as “TGF-β signaling in thyroid cells” and “Type 2 Papillary Renal Cell Carcinoma”, which are linked to epithelial-mesenchymal transition and metastatic potential [27–29]. Interestingly, while prior study has reported that low PBX1 expression is associated with enhanced CRC proliferation and invasion, and that PBX1 overexpression can suppress tumor growth [13], our data revealed a paradoxical enrichment of apoptosis-related signaling upon *PBX1* knockdown. This suggests that although *PBX1* overexpression may inhibit proliferative capacity in some contexts, it could simultaneously promote tumor cell apoptosis. Therefore, strategies aiming to suppress tumor growth via *PBX1* overexpression may carry the unintended consequence of inhibiting tumor cell apoptosis, potentially leading to an accumulation of quiescent, less active, and drug-resistant tumor cells. These results underscore the need for further investigation into the relationship between *PBX1* expression and apoptotic regulation in cancer.

To further investigate the regulatory mechanisms underlying the transcriptional changes observed upon *PBX1* knockdown, we performed a CUT&Tag assay in HCT116 cells using a PBX1-specific antibody to map genome-wide PBX1 binding sites. Given PBX1’s established role as a transcription factor, we aimed to identify genes that are not only differentially expressed following *PBX1* depletion but also directly bound by PBX1 on chromatin. Figure 4C shows that PBX1 binding peaks are predominantly located in intergenic regions (40.29%) and intronic regions (39.94%), while ~14.49% of binding events occur near transcription start sites (TSS), suggesting PBX1 binds both distal and proximal regulatory elements.

To determine the regulatory context of PBX1-bound regions, we analyzed histone modification signals at these loci, focusing on H3K4me3, H3K27ac, and H3K4me1-canonical markers for promoters and enhancers. As shown in the top of Fig. 4D, the majority of PBX1 binding sites exhibited strong H3K4me3 and H3K27ac enrichment, along with relatively low H3K4me1 levels. This epigenetic signature is characteristic of active promoters, whereas we observed no clear evidence of enhancer-like regions defined by high H3K4me1 and H3K27ac but low H3K4me3.

Based on this chromatin landscape, we annotated PBX1 binding peaks to nearby promoters and compiled a list of 701 genes with PBX1 occupancy at their promoter regions across three independent biological replicates (Fig. 4E). To further narrow down functionally relevant targets, we intersected this list with the 89 differentially expressed genes identified from RNA-seq analysis. This integrative approach yielded four high-confidence candidate genes that are both transcriptionally regulated by PBX1 and directly bound at their promoters: *DUSP5*, *AP3S1*, *PLK2*, and *BCL2L1* (Fig. 4F).

Among the four candidate genes identified—*DUSP5, AP3S1, PLK2*, and *BCL2L1*, several have been previously linked to tumor-related processes. *DUSP5* has been shown to regulate MAPK/ERK signaling and influence tumor cell proliferation and migration [30, 31]. *PLK2* is involved in cell cycle regulation [32, 33], while *AP3S1* remains less well characterized in cancer biology. *BCL2L1*, which encodes the anti-apoptotic protein Bcl-xL, is frequently upregulated in tumors and associated with enhanced cell survival [34–36]. In addition, Fig. 4G shows that *BCL2L1* displayed the most significant change, with an adjusted p-value of 6.253 × 10^-8^, indicating it is highly responsive to *PBX1* depletion. In parallel, CUT&Tag profiling confirmed that PBX1 is strongly enriched at the promoter region of *BCL2L1* (Fig. 4H), supporting the likelihood that *BCL2L1* is a direct transcriptional target of PBX1 in colorectal cancer cells.

### PBX1-driven upregulation of *BCL2L1* promoter activity in CRC cells

**Fig. 5:**
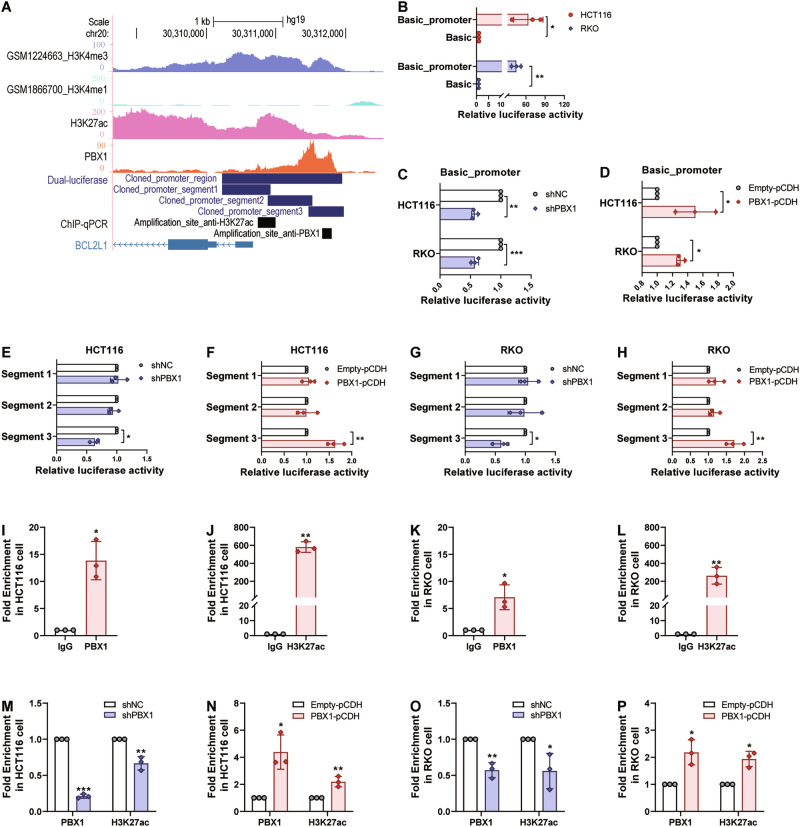
*PBX1* activates *BCL2L1* promoter activity by direct binding and chromatin activation. **A** Diagram of the *BCL2L1* promoter region used for luciferase reporter assay and ChIP-qPCR. **B** Luciferase reporter activity of the full-length *BCL2L1* promoter in HCT116 and RKO cells. **C** Luciferase activity of the *BCL2L1* promoter after *PBX1* knockdown in HCT116 and RKO cells. **D** Luciferase activity of the *BCL2L1* promoter after *PBX1* overexpression in HCT116 and RKO cells. **E**–**H** Fragment-specific luciferase assays in HCT116 and RKO cells. *PBX1* knockdown **E**, **G** markedly reduced the luciferase activity driven by Fragment 3, while *PBX1* overexpression **F**, **H** significantly enhanced the activity of Fragment 3. **I**–**L** ChIP-qPCR analysis of PBX1 and H3K27ac enrichment at the *BCL2L1* promoter region in HCT116 and RKO cells. **M**, **O** *PBX1* knockdown reduced the enrichment of PBX1 and H3K27ac at the *BCL2L1* promoter in HCT116 and RKO cells. **N**, **P** *PBX1* overexpression increased PBX1 and H3K27ac enrichment at the *BCL2L1* promoter. Significance for all data was determined by the independent samples t-test. Data are shown as mean ± S.D., *n* = 3. *P < 0.05, **P < 0.01, ***P < 0.001.

To elucidate the regulatory mechanism by which *PBX1* modulates *BCL2L1* expression, we performed ChIP-qPCR and dual-luciferase reporter assays. As illustrated in Fig. 5A, we first cloned a ~ 1719 bp fragment of the *BCL2L1* promoter region into the pGL3-Basic vector to construct a full-length promoter reporter plasmid. Based on the CUT&Tag data indicating *PBX1* binding intensity, this promoter region was further subdivided into three overlapping fragments (Fragments 1, 2, and 3), which were individually cloned into the pGL3-Basic vector to assess *PBX1*’s effect on discrete regulatory elements.

Luciferase reporter assays demonstrated that the full-length *BCL2L1* promoter construct exhibited robust promoter activity in both HCT116 and RKO cells, with approximately 65-fold and 45-fold activation, respectively, compared to the empty vector control (Fig. 5B). Knockdown of *PBX1* via shRNA significantly reduced the promoter activity in both cell lines (Fig. 5C), whereas stable overexpression of *PBX1* led to a marked increase in promoter activity (Fig. 5D).

To further localize the critical *PBX1*-responsive region within the promoter, we assessed the activity of the three truncated promoter fragments. In HCT116 cells, knockdown of *PBX1* resulted in a significant decrease in luciferase activity driven by Fragment 3, while Fragments 1 and 2 showed minimal changes (Fig. 5E). Conversely, PBX1 overexpression significantly enhanced the activity of Fragment 3 (Fig. 5F). Similar results were observed in RKO cells, where *PBX1* knockdown suppressed (Fig. 5G) and *PBX1* overexpression enhanced (Fig. 5H) the activity of Fragment 3, indicating that this region contains a critical PBX1-responsive element.

To verify the binding of PBX1 to the *BCL2L1* promoter in vivo and assess its impact on chromatin activation, we performed ChIP-qPCR targeting PBX1 and H3K27ac. In the ChIP-qPCR assay, we assessed the enrichment of PBX1 at this region, as well as its impact on chromatin activation status, marked by the histone modification H3K27ac [37]. Based on previous studies showing that transcription factor binding sites and histone modifications such as H3K27ac often occur in adjacent but non-overlapping regions within regulatory domains [38, 39], we designed ChIP-qPCR primers to independently capture the peak enrichment sites for PBX1 and H3K27ac, respectively, according to our CUT&Tag and luciferase reporter assays results.

As shown in Fig. 5I–L, both PBX1 and H3K27ac were significantly enriched at the *BCL2L1* promoter in HCT116 and RKO cells, with enrichment levels being notably higher in HCT116 cells. PBX1 knockdown led to a substantial reduction in PBX1 and H3K27ac occupancy at the promoter region (Fig. 5M and O), whereas PBX1 overexpression increased their enrichment (Fig. 5N and P), suggesting that PBX1 binding facilitates chromatin activation of the *BCL2L1* promoter through H3K27 acetylation.

Collectively, these results demonstrate that PBX1 directly binds to a critical enhancer region within the *BCL2L1* promoter, enhancing its transcriptional activity by modulating local chromatin accessibility.

### PBX1-mediated upregulation of Bcl-xL suppresses apoptosis

To further clarify the regulatory effect of *PBX1* on *BCL2L1* expression, we conducted both knockdown and overexpression experiments in HCT116 and RKO colorectal cancer cells. Specifically, we assessed how changes in *PBX1* expression influence the two major *BCL2L1* transcript variants, which encode the protein isoforms Bcl-xL and Bcl-xS. As described in the Introduction, Bcl-xL functions as an anti-apoptotic protein, whereas Bcl-xS promotes apoptosis.

**Fig. 6:**
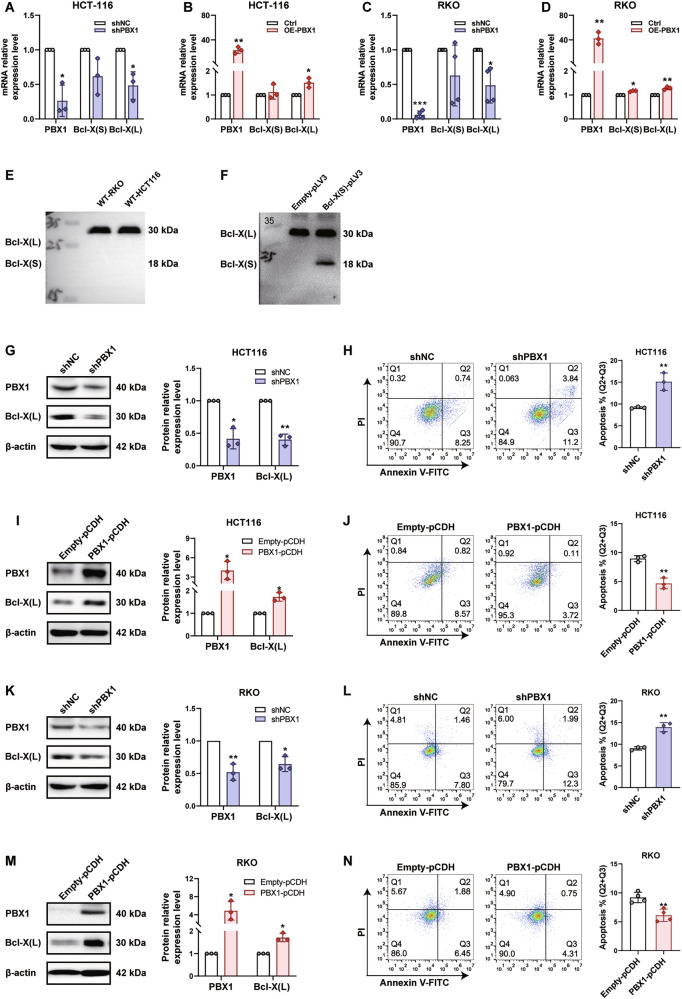
*PBX1* regulates isoform-specific expression of *BCL2L1* and modulates apoptosis. **A**, **C** *PBX1* knockdown decreases Bcl-xL mRNA in HCT116 (**A**) and RKO (**C**) cells; **B**, **D** overexpression increases Bcl-xL mRNA in HCT116 (**B**) and RKO (**D**) cells, with little change in Bcl-xS. **E** Western blot confirms predominant expression of Bcl-xL in CRC cells; **F** Bcl-xS detectable only upon exogenous overexpression. **G**, **H** *PBX1* knockdown reduces Bcl-xL protein and increases apoptosis in HCT116 cells; **I**, **J** Overexpression of *PBX1* increases Bcl-xL and decreases apoptosis in HCT116 cells. **K**, **L** *PBX1* knockdown reduces Bcl-xL protein and increases apoptosis in RKO cells; **M**, **N** Overexpression of *PBX1* increases Bcl-xL and decreases apoptosis in RKO cells. Significance for all data was determined by the independent samples t-test. Data are shown as mean ± S.D., *n* >= 3. *P < 0.05, **P < 0.01.

We therefore measured the mRNA levels of Bcl-xL and Bcl-xS separately. As shown in Fig. 6A and C, *PBX1* knockdown significantly reduced the expression of Bcl-xL, while Bcl-xS levels remained unchanged. Conversely, overexpression of *PBX1* led to a marked increase in Bcl-xL mRNA, with only a modest upregulation of Bcl-xS observed in RKO cells (Fig. 6B and D). These results suggest that PBX1 predominantly regulates the expression of the anti-apoptotic isoform Bcl-xL at the mRNA level in colorectal cancer cells. Consistent with the mRNA-level findings, our long-term observations from repeated WB experiments revealed that CRC cells predominantly express the Bcl-xL protein isoform (~30 kDa). Even under overexposed blot conditions, the Bcl-xS isoform (~ 18 kDa) was undetectable in endogenous settings (Fig. 6E). Only upon exogenous overexpression of Bcl-xS from a plasmid could we detect the corresponding protein band in HCT116 cells (Fig. 6F). Taken together, these results suggest that in CRC cells, *PBX1* primarily regulates the Bcl-xL isoform of *BCL2L1*.

Given the predominance of the Bcl-xL isoform in CRC cells, subsequent protein-level analyses focused specifically on Bcl-xL. As shown in Fig. 6G and H, *PBX1* knockdown in HCT116 cells led to a reduction in Bcl-xL protein levels, accompanied by an increase in apoptotic cells. Conversely, *PBX1* overexpression resulted in elevated Bcl-xL protein expression and a decrease in apoptosis in HCT116 (Fig. 6I and J). Similar results were observed in RKO cells, where *PBX1* overexpression upregulated Bcl-xL protein and reduced apoptosis, while *PBX1* knockdown produced the opposite effect (Fig. 6K–N). Together, these findings indicate that *PBX1* modulates apoptosis in colorectal cancer cells primarily through regulation of the anti-apoptotic isoform Bcl-xL.

To further explore the potential feedback mechanism between *PBX1* and *BCL2L1*, we investigated whether *BCL2L1* regulates *PBX1* expression or activity. We overexpressed or silenced *BCL2L1* in HCT116 and RKO colorectal cancer cells and assessed *PBX1* expression at both the mRNA and protein levels. As shown in Supplementary Fig. S1A-D, neither silencing nor overexpression of *BCL2L1* significantly altered *PBX1* mRNA levels. Additionally, Western blot analysis (Supplementary Fig. S1E-H) revealed that *PBX1* protein expression remained unchanged upon *BCL2L1* modulation in both cell lines. These results suggest that *BCL2L1* does not significantly regulate *PBX1* expression or activity in colorectal cancer cells, indicating that the regulation of *PBX1* by *BCL2L1* is unidirectional in this context.

### Functional interaction between PBX1 and *BCL2L1* in regulating apoptosis and tumor growth

**Fig. 7:**
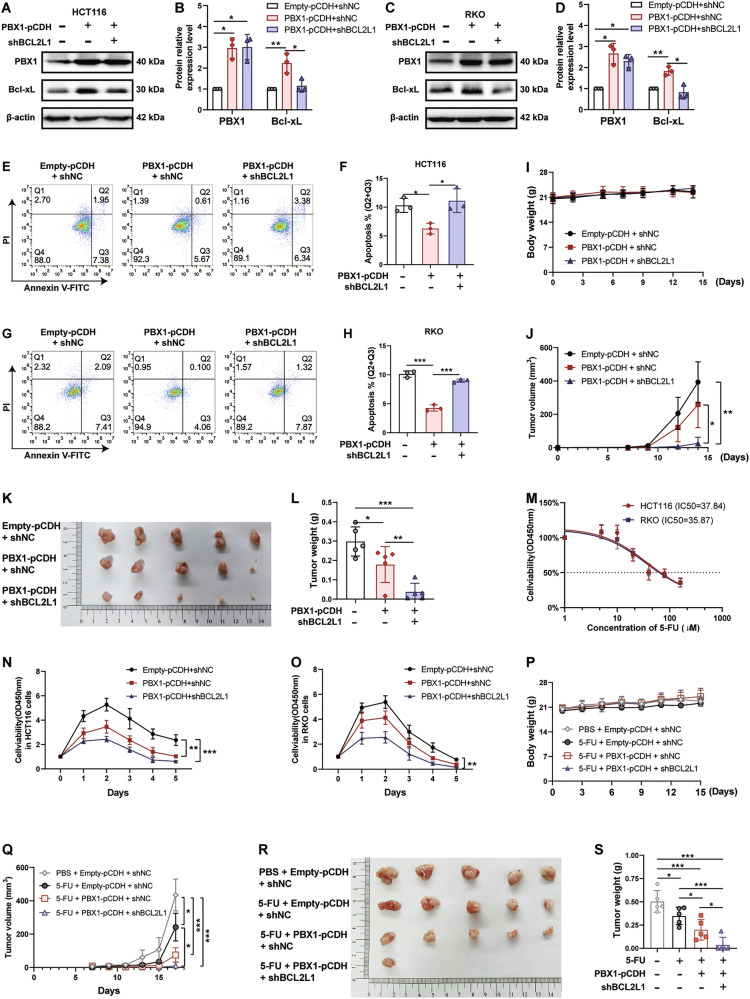
Functional validation of the *PBX1*–*BCL2L1* regulatory axis. **A**, **B** Western blot and quantification showing that *PBX1* overexpression increases Bcl-xL protein levels in HCT116 cells, while *BCL2L1* knockdown reduces Bcl-xL expression. **C**, **D** Similar analysis in RKO cells confirms that *PBX1* upregulates Bcl-xL, which is suppressed upon *BCL2L1* knockdown. **E**, **F** Flow cytometry analysis of apoptosis in HCT116 cells shows that *PBX1* overexpression reduces apoptosis, whereas *BCL2L1* knockdown restores apoptotic levels. **G**, **H** Consistent apoptotic rescue effect observed in RKO cells following *BCL2L1* knockdown in the context of *PBX1* overexpression. **I**–**L** *PBX1* overexpression and *BCL2L1* Knockdown in Nude Mice: **I** Body weight of mice measured throughout the experimental period. **J** Tumor volume growth curves during the experiment. **K** Images of tumors excised from mice at the experimental endpoint. **L** Quantification of tumor weight. **M** Dose-response curves of HCT116 and RKO cells treated with 5-FU for 24 h. **N**, **O** Cell viability following 10 µM 5-FU treatment in HCT116 and RKO. **P**–**S** *PBX1* overexpression and *BCL2L1* Knockdown with 5-FU Treatment in Nude Mice: **P** Body weight of mice measured throughout the experimental period. **Q** Tumor volume growth curves during the experiment. **R** Images of tumors excised from mice at the experimental endpoint. **S** Quantification of tumor weight. Data are shown as mean ± S.D., *n* >= 3. Statistical significance was assessed using one-way ANOVA with LSD post-hoc test. *P < 0.05, **P < 0.01, ***P < 0.001.

To further explore the functional relationship between PBX1 and *BCL2L1* in regulating tumor cell fate, we examined the effects of *BCL2L1* knockdown in the context of *PBX1* overexpression. As shown in Fig. 7A and B, overexpression of *PBX1* in HCT116 cells led to a marked increase in Bcl-xL protein levels, while silencing *BCL2L1* (shBCL2L1) effectively diminished Bcl-xL expression, confirming that *PBX1* enhances Bcl-xL expression through transcriptional upregulation. Similar results were observed in RKO cells (Fig. 7C and D), indicating that this regulatory mechanism is consistent across CRC models.

Apoptosis assays (Fig. 7 E-H) demonstrated that *PBX1* overexpression alone significantly suppressed apoptosis in HCT116 and RKO cells. However, this anti-apoptotic effect was reversed upon *BCL2L1* knockdown, with apoptosis levels restored to baseline, indicating that *PBX1* modulates apoptosis predominantly through *BCL2L1*-dependent Bcl-xL upregulation.

To validate these findings in vivo, we established xenograft models in nude mice with HCT116 cells and divided them into three groups: control (empty-pCDH + shNC), *PBX1* overexpression (PBX1-pCDH + shNC), and *PBX1* overexpression combined with *BCL2L1* knockdown (PBX1-pCDH + shBCL2L1). Figure 7I shows that there were no significant differences in body weight across groups during the experiment, suggesting no overt toxicity from the treatments. Tumor volume monitoring over time (Fig. 7J) confirmed that tumors in the *PBX1* overexpression combined with *BCL2L1* knockdown group grew significantly slower than those in the other groups, while no significant difference was observed between the control and *PBX1*-overexpressing groups alone. Tumor images (Fig. 7K) and corresponding tumor weights (Fig. 7L) further supported this trend: the control group had the heaviest tumors, followed by the *PBX1* overexpression group, with the *PBX1* overexpression combined with *BCL2L1* knockdown group displaying the lowest tumor burden.

To evaluate the impact of this regulatory axis on chemotherapy sensitivity, we treated HCT116 and RKO cells with increasing doses of 5-fluorouracil (5-FU) and assessed cell viability. As shown in Fig. 7M, dose-response curves revealed IC50 values of 37.84 µM for HCT116 cells and 35.87 µM for RKO cells after 24-hour exposure. Based on these results, a 10 µM treatment concentration was selected, representing approximately one-quarter to one-half of the IC50 [40]. At this concentration, *PBX1* overexpression alone significantly reduced cell viability in HCT116 cells (Fig. 7N), suggesting enhanced drug sensitivity. Importantly, combined *PBX1* overexpression and *BCL2L1* knockdown led to an even more pronounced reduction in cell viability, highlighting a synergistic inhibitory effect and reinforcing the relevance of the *PBX1*–*BCL2L1* axis under chemotherapeutic stress. A similar trend was observed in RKO cells (Fig. 7O).

In vivo, we performed a series of experiments using HCT116 cells stably transfected with Empty-pCDH+shNC, PBX1-pCDH+shNC, or PBX1-pCDH+shBCL2L1 constructs, and treated the mice with 5-FU. The four treatment groups were: (i) Empty-pCDH+shNC treated with PBS (control), (ii) Empty-pCDH+shNC treated with 5-FU, (iii) PBX1-pCDH+shNC treated with 5-FU, and (iv) PBX1-pCDH +shBCL2L1 treated with 5-FU. As shown in Fig. 7P, no significant differences in body weight were observed across the groups, suggesting no significant systemic toxicity. Tumor volume (Fig. 7Q) was significantly smaller in the 5-FU treatment groups compared to the PBS treatment group, with the PBX1-pCDH+shBCL2L1 group showing the smallest tumors, indicating enhanced chemosensitivity. Tumor weight data (Fig. 7R and S) further confirmed that tumors in the PBX1-pCDH +shBCL2L1 group were significantly lighter than those in other groups, reinforcing the synergistic effect of *PBX1* overexpression and *BCL2L1* knockdown in reducing tumor burden under chemotherapy.

These results indicate that while *PBX1* overexpression alone moderately suppresses tumor growth, the concurrent silencing of *BCL2L1* substantially enhances this effect, supporting a functional interaction between *PBX1* and *BCL2L1* in regulating tumor growth in CRC models.

## Discussion

Given the central role of apoptosis evasion in cancer progression and therapeutic resistance, our study explores the potential involvement of the *PBX1*–*BCL2L1* axis contributes to apoptosis suppression and modulates tumor cell fate in CRC. We observed hat *PBX1* overexpression inhibits CRC cell proliferation, migration, and invasion, suggesting a tumor-suppressive function in this context. However, *PBX1* overexpression concurrently reduces apoptosis, potentially allowing cells to enter a quiescent, drug-resistant state. This dual effect complicates the therapeutic use of *PBX1* overexpression alone, as it may restrict the efficacy of pro-apoptotic treatments.

Our analysis of public datasets (TCGA and Oncomine) and patient samples confirmed that *PBX1* is consistently downregulated in CRC tissues and cell lines, supporting previous findings that *PBX1* often plays a tumor-suppressive role in solid tumors [13]. Notably, analysis of *PBX1* expression across different CRC subtypes revealed a subtype-dependent prognostic significance. In CMS1 (immune subtype), low *PBX1* expression was associated with poorer survival, suggesting a potential link between *PBX1* downregulation and immune evasion, which may contribute to worse outcomes in this subtype. Conversely, in CMS2 (epithelial subtype), high *PBX1* expression was correlated with better survival, indicating that *PBX1* may help maintain epithelial differentiation and suppress malignant proliferation. These findings suggest a context-dependent role of *PBX1* across CRC subtypes, although the underlying mechanisms require further investigation.

Mechanistically, transcriptomic and epigenomic profiling revealed *BCL2L1* as a direct transcriptional target of PBX1. PBX1 binds the *BCL2L1* promoter and enhances its transcription, supported by increased H3K27ac enrichment and luciferase reporter activation. These results align with previous studies that demonstrate PBX1’s ability to regulate chromatin accessibility and gene expression at key loci [41]. Further isoform-specific analysis showed that PBX1 primarily upregulates Bcl-xL, the anti-apoptotic isoform, in a splicing environment biased toward Bcl-xL. This was validated by qPCR and western blotting, which detected endogenous Bcl-xL but not Bcl-xS in CRC cells. These findings suggest that PBX1 increases the overall transcription of *BCL2L1*, and in the presence of a cell-intrinsic splicing bias, promotes Bcl-xL expression, contributing to apoptosis resistance.

Functional assays confirmed that *PBX1* knockdown decreased Bcl-xL levels and increased apoptosis, while overexpression of *PBX1* elevated Bcl-xL and reduced apoptotic cell death. These findings align with prior studies highlighting the oncogenic role of Bcl-xL in tumor survival and drug resistance [42]. Importantly, knocking down *BCL2L1* in *PBX1*-overexpressing cells reversed *PBX1*’s anti-apoptotic effect, indicating that Bcl-xL functions as a key downstream mediator of PBX1-dependent survival signaling. While *PBX1* overexpression modestly reduced proliferation, it enhanced cell survival, a phenotype that was abolished when *BCL2L1* was knocked down. This finding supports a model in which *PBX1* may contribute to the persistence of non-proliferative, apoptosis-resistant tumor cells, which could potentially influence treatment response.

Functional assays further supported this regulatory preference: *PBX1* knockdown decreased Bcl-xL and significantly increased apoptosis, while *PBX1* overexpression elevated Bcl-xL expression and suppressed apoptotic activity. Importantly, functional interaction experiments in *PBX1*-overexpressing cells demonstrated that *BCL2L1* knockdown reversed the anti-apoptotic effect of *PBX1*, further supporting that Bcl-xL is a key functional effector downstream of *PBX1*. In addition, while *PBX1* overexpression modestly suppressed proliferation, it simultaneously enhanced cell survival—an effect that was abrogated by *BCL2L1* knockdown. This dual effect suggests that *PBX1* may promote the persistence of non-proliferative, apoptosis-resistant tumor cells, a phenomenon that warrants further investigation in therapeutic contexts.

To assess the clinical relevance of this axis, we evaluated how *PBX1* and *BCL2L1* influence 5-FU chemotherapy response. *PBX1* overexpression reduced cell viability under 5-FU exposure, and the combination of *PBX1* overexpression with *BCL2L1* knockdown further enhanced 5-FU cytotoxicity. These results suggest that targeting *BCL2L1* may improve the therapeutic efficacy of *PBX1*-based modulation. Similar patterns were observed in RKO cells, reinforcing the generalizability of the *PBX1—BCL2L1* axis in CRC.

In vivo, xenograft experiments confirmed that *PBX1* overexpression suppresses tumor growth, and this effect was significantly augmented by *BCL2L1* knockdown. Tumor volume and weight were lowest in the *PBX1* overexpression combined with *BCL2L1* knockdown group, supporting a synergistic effect in vivo. These findings indicate the cooperative role of *PBX1* and *BCL2L1* in modulating tumorigenesis.

Beyond *BCL2L1*, we identified additional *PBX1*-regulated targets such as *DUSP5* and *PLK2*, which are known to inhibit MAPK signaling and cell cycle progression, respectively [31, 32]. These targets likely contribute to the anti-proliferative function of *PBX1* and further establish its multifaceted role in CRC suppression.

**Fig. 8:**
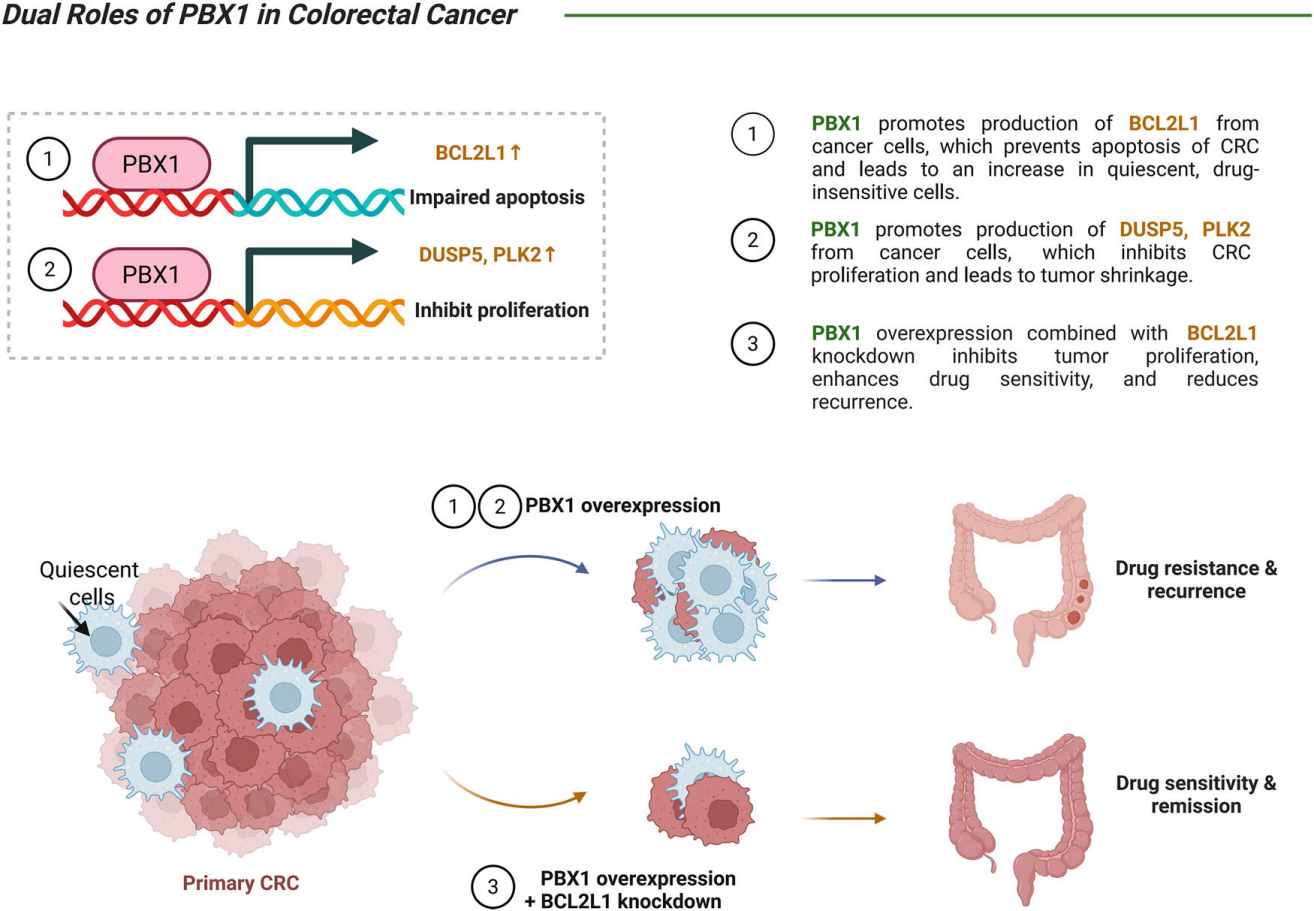
Working model of *PBX1* dual regulatory roles in colorectal cancer. *PBX1* activates *BCL2L1* transcription to promote expression of anti-apoptotic Bcl-xL, suppressing apoptosis and promoting the survival of quiescent or drug-resistant cells. Simultaneously, *PBX1* induces tumor suppressor genes (*DUSP5* and *PLK2*), which inhibit proliferation. Therapeutically, co-targeting *PBX1* and *BCL2L1* may overcome this balance and enhance treatment efficacy. Created with BioRender.com.

In summary, we propose a working model in which *PBX1* simultaneously promotes anti-proliferative and anti-apoptotic pathways in CRC (Fig. 8). *PBX1* directly enhances *BCL2L1* transcription, leading to Bcl-xL–mediated apoptotic resistance. Concurrently, *PBX1* activates tumor-suppressive genes like *DUSP5* and *PLK2*, inhibiting proliferation. This dual regulation creates a state in which tumor cells are growth-arrested yet resistant to apoptosis, potentially contributing to therapy resistance. Disrupting this balance by combining *PBX1* overexpression with *BCL2L1* knockdown effectively inhibits both proliferation and survival, thereby improving treatment efficacy. Overall, the *PBX1*–*BCL2L1* axis represents a regulatory pathway that may influence tumor progression and therapeutic response in colorectal cancer, although further translational studies are required to determine its clinical applicability.

Nevertheless, several limitations of this study should be acknowledged. Although xenograft experiments support a functional interaction between *PBX1* and *BCL2L1*, therapeutic delivery strategies targeting this axis were not evaluated. Development of clinically applicable interventions will require extensive pharmacological and safety validation beyond the scope of this work. In addition, the current findings are primarily based on in vitro systems and immunodeficient mouse models. Further validation in immune-competent and clinically relevant models will help clarify the broader biological and translational significance of the *PBX1–BCL2L1* axis in colorectal cancer.

## Materials and methods

### Patients and tissues

This study examined 50 cases of colonic adenocarcinoma and corresponding adjacent normal colonic mucosa, collected from patients who underwent surgical treatment at Shantou Central Hospital (Guangdong, China) between 2020 and 2022. These fresh tissue samples were used for quantitative PCR (qPCR) and Western blot (WB) analysis. Patients who had received radiotherapy or chemotherapy prior to surgery were excluded. Additionally, immunohistochemical (IHC) analysis was performed on a separate cohort of 40 cases collected between 2015 and 2025, with 20 cases in Stage I-II and 20 cases in Stage III-IV based on the TNM staging system. These samples were used for IHC to examine PBX1 expression. All tissues were paraffin-embedded and diagnosed independently by two pathologists. Ethical approval was obtained from the local ethics committee (Shantou University Medical College, SUMC-2020-14), and informed consent was acquired.

### Cell lines and culture conditions

Human colorectal cancer cell lines, including HCT116, HT-29, LoVo, RKO, SW480, and SW620, and the normal colon cell line CCD-18Co, were obtained from ATCC (Manassas, VA, USA) or the Shanghai Cell Bank, Chinese Academy of Sciences (LoVo). Cells were maintained in cell line-specific media as follows: McCoy’s 5 A Medium (for HCT116 and HT-29; Gibco, Cat. No. 16600082); F-12K Nutrient Mixture (for LoVo; Gibco, Cat. No. 21127022); Minimum Essential Medium (MEM) supplemented with 2 mM L-glutamine and 1 mM sodium pyruvate (for RKO; Gibco, Cat. No. 11380037); Leibovitz’s L-15 Medium (for SW480 and SW620; Gibco, Cat. No. 11415064); MEM supplemented with 2 mM L-glutamine, 1 mM sodium pyruvate, and 1 × non-essential amino acids (NEAA) (for CCD-18Co; Gibco, Cat. No. 11140050). All media were supplemented with 10% fetal bovine serum (FBS) (Gibco, Cat. No. 10099141), and cells were incubated at 37 °C in a humidified atmosphere containing 5% CO₂ (except for L-15, which was cultured in atmospheric air).

### RNA extraction, cDNA synthesis, and qPCR

Total RNA was extracted using RNAiso Plus (Takara Bio Inc., Shiga, Japan; Cat. No. 9109). First-strand cDNA synthesis was performed using the HiScript II Q RT SuperMix for qPCR (Vazyme Biotech Co., Ltd., Nanjing, China; Cat. No. R223-01). Quantitative PCR was conducted with the ChamQ SYBR qPCR Master Mix (Vazyme Biotech Co., Ltd., Nanjing, China; Cat. No. Q331) on an ABI 7500 Real-Time PCR System (Applied Biosystems, Foster City, CA, USA). β-actin was used as an internal control. Primer sequences are listed in Supplementary Table S2.

### Western blot analysis

Proteins were resolved on 10% SDS-PAGE gels and transferred onto PVDF membranes (Sigma-Aldrich, St. Louis, MO, USA; Cat. No. ISEQ00010). Membranes were incubated with primary antibodies followed by HRP-conjugated secondary antibodies. Signals were visualized using enhanced chemiluminescent detection reagents (Yeasen Biotechnology, Shanghai, China; Cat. No. 36208ES76). The primary antibodies used included: Rabbit anti-PBX1 (Proteintech Group, Wuhan, China; Cat. No. 18204-1-AP; dilution 1:500), Mouse anti-Bcl-xS/L (Santa Cruz Biotechnology, Dallas, TX, USA; Cat. No. sc-271121; dilution 1:200), Mouse anti-β-actin (Proteintech Group, Wuhan, China; Cat. No. 66009-1-Ig; dilution 1:10000). The secondary antibodies used were: HRP-conjugated goat anti-mouse IgG (Thermo Fisher Scientific, Waltham, MA, USA; Cat. No. 31430; dilution 1:5000), HRP-conjugated goat anti-rabbit IgG (Thermo Fisher Scientific, Waltham, MA, USA; Cat. No. 31460; dilution 1:10000).

### Database analysis

*PBX1* expression in CRC was assessed using TCGA and Oncomine databases. TCGA RNA-seq data were analyzed using DESeq2 (v1.46.0), identifying significantly differentially expressed genes (adjusted *p* < 0.05, |fold change | ≥ 2). Oncomine Database Analysis: We queried the database to analyze *PBX1* mRNA expression in CRC patients. By comparing multiple datasets provided by Oncomine (www.oncomine.org, accessed on 1 March 2021), downloaded in March 2021, and data were analyzed with thresholds of *p* < 0.0001 and fold change > 2. TNMplot analysis: *PBX1* expression in normal, primary, and metastatic colorectal tissues was analyzed using the TNMplot online database (https://tnmplot.com/analysis/, accessed on 24 October 2025) [43]. The comparison between normal mucosa, primary tumor, and metastatic lesions was performed using the Kruskal-Wallis test followed by Dunn’s multiple comparison test. R2 database analysis: *PBX1* expression and its prognostic relevance across consensus molecular subtypes (CMS1-4) were assessed using the R2 Genomics Analysis and Visualization Platform (Tumor Colon (CMS) - Guinney-3232-custom-ccrcst1 dataset; R2: Genomics Analysis and Visualization Platform, http://r2.amc.nl) [44]. Subtype comparisons were performed using one-way ANOVA with LSD post hoc test. Survival analysis was conducted using Kaplan-Meier curves, with cutoff mode set to “curtain” to determine the optimal threshold for *PBX1* expression in each CMS subtype.

### Cell transfections

For stable gene modulation, lentiviral transduction was employed to achieve *PBX1* overexpression and shRNA-mediated knockdown. Lentiviral particles were generated by co-transfecting packaging plasmids and expression vectors into HEK293T cells using standard calcium phosphate or lipid-based methods. Target CRC cells were infected with viral supernatants in the presence of 8 µg/mL polybrene (Sigma-Aldrich, St. Louis, MO, USA; Cat. No. H9268) for 24 h. Infected cells were selected using puromycin (1 µg/mL; Sangon Biotech, Shanghai, China; Cat. No. A610593) and/or blasticidin (8 µg/mL; Beyotime, Shanghai, China; Cat. No. ST018), or sorted by flow cytometry when applicable.

The *PBX1* overexpression plasmid was constructed by inserting the full-length coding sequence of *PBX1* (NM_001204961.2) into the pCDH-CMV-EF1-copGFP lentiviral backbone (System Biosciences, Palo Alto, CA, USA). Additionally, two other plasmids were constructed in the pLV3-CMV-3 × FLAG-EF1a-CopGFP-Neo backbone (Shanghai Bioersn Biotechnology Co., Ltd., Shanghai, China): Bcl-X(S) (NM_001191.4) and Bcl-X(L) (NM_001317919.2). shRNA plasmids were obtained from GenePharma (Suzhou, China): shPBX1 was cloned into the LV3 (H1/GFP&Puro) backbone, targeting sequence: 5’-GTGGAGCATTCAGATTACA-3’, and shBCL2L1 was cloned into the pLKO. 1-blast backbone, targeting sequence: 5’-CCCTACCTGATTGGTGCAA-3’.

### RNA sequencing and data processing

Total RNA from *PBX1*-knockdown and control HCT116 cells was sequenced by BGI Genomics (Shenzhen, China) using the BGISEQ platform. Reads were mapped to GRCh38 using RSEM, and differential expression analysis was conducted using DESeq2 (v1.46.0). Gene set enrichment analysis (GSEA, v4.3.3) was performed with the MSigDB collection c2.cp.wikipathways.v2024.1. NES and FDR < 0.25 were considered significant.

### CUT&Tag assay and data processing

CUT&Tag assays were conducted using the Hyperactive Universal CUT&Tag Assay Kit for Illumina Pro (Vazyme, TD904; Vazyme Biotech, Nanjing, China), with a mild formaldehyde crosslinking step added prior to cell permeabilization. Library construction was performed via Tn5 tagmentation followed by PCR amplification using the reagents provided in the kit. Final libraries were sequenced on the BGISEQ platform (BGI Genomics, Shenzhen, China). Raw reads were aligned to the human genome using Bowtie2 (v2.3.5. 1), and peak calling was performed with SEACR (Sparse Enrichment Analysis for CUT&RUN). Peak annotation and motif enrichment analyses were carried out using HOMER (v4. 11).

CUT&Tag experiments targeting PBX1 and H3K27ac were performed in-house. The following primary antibodies were used: anti-PBX1 (Abnova, H00005087-M01, mouse monoclonal, 1:50; Taipei, Taiwan), anti-H3K27ac (Invitrogen, 720096, rabbit polyclonal, 1:50; Carlsbad, CA, USA). Public ChIP-seq datasets for H3K4me3 (GEO: GSM1224663) and H3K4me1 (GEO: GSM1866700) were downloaded from the GEO database for comparative chromatin landscape profiling.

### Cell proliferation assay

Cell proliferation was assessed using the Cell Counting Kit-8 (CCK-8; MedChemExpress, HY-K0301; Monmouth Junction, NJ, USA) following the manufacturer’s instructions. Cells were seeded in 96-well plates at appropriate densities according to cell line characteristics. Absorbance at 450 nm was measured daily using a microplate reader to generate cell growth curves.

### Colony formation assay

For colony formation assays, cells were seeded in 6-well plates at a density of 300 cells per well and cultured for 2 weeks. Colonies were fixed with 4% paraformaldehyde and stained with crystal violet (Beyotime, C0121; Shanghai, China) for 30 minutes at room temperature. The colony-covered area was quantified using ImageJ software (version 1.46r; NIH, Bethesda, MD, USA) and compared to the control group.

### Transwell migration and invasion assay

For cell migration and invasion assays, Transwell chambers (8-µm pore size; BD Falcon, 35309724; Franklin Lakes, NJ, USA) were used. For invasion assays, the upper chambers were pre-coated with Matrigel (BD Biosciences, 356234; San Jose, CA, USA). Cells were serum-starved for 12 h, seeded into the upper chambers in serum-free medium, and allowed to migrate toward medium containing 20% fetal bovine serum in the lower chambers. After 24 h of incubation, cells on the lower surface were fixed with 4% paraformaldehyde and stained with crystal violet (Beyotime, C0121; Shanghai, China) for 30 min at room temperature.

### Cell apoptosis assay

Apoptosis was evaluated using the APC Annexin V Apoptosis Detection Kit (Tonbo Biosciences, 20-6410-KIT; San Diego, CA, USA) according to the manufacturer’s instructions. Cells were stained with APC Annexin V and 7-AAD, and analyzed by flow cytometry using a Celula Sparrow2040 flow cytometer (Celula Inc., Shanghai, China). The percentages of early and late apoptotic cells were quantified.

### Chromatin immunoprecipitation (ChIP)

Chromatin immunoprecipitation (ChIP) was performed using the EZ-ChIP™ Chromatin Immunoprecipitation Kit (Sigma-Aldrich, 17-611; St. Louis, MO, USA) according to the manufacturer’s instructions, as previously described [45]. Immunoprecipitated DNA was analyzed by quantitative PCR, and results were normalized to input DNA. The following primary antibodies were used: anti-PBX1 (Abnova, H00005087-M01, mouse monoclonal, 1:50; Taipei, Taiwan), anti-H3K27ac (Invitrogen, 720096, rabbit polyclonal, 1:50; Carlsbad, CA, USA), mouse IgG control (Millipore, 12-371B, 1:50; Burlington, MA, USA), rabbit IgG control (Millipore, CS200581, 1:50; Burlington, MA, USA). Primer sequences used for qPCR analysis are listed in Supplementary Table S3.

### Dual-luciferase reporter assay

The BCL2L1 promoter (~1719 bp) and its truncated fragments (Fragment 1: 695 bp; Fragment 2: 638 bp; Fragment 3: 503 bp) were amplified using specific primers (sequences listed in Supplementary Table S4) and cloned into the pGL3-Basic vector (Promega, Madison, WI, USA) at the HindIII restriction site using the ClonExpress II One Step Cloning Kit (Vazyme, C116; Nanjing, China). For luciferase activity measurements, HCT116 and RKO cells were co-transfected with the constructed firefly luciferase plasmids and Renilla luciferase internal control plasmid (pRL-SV40, Promega, Madison, WI, USA) at a molar ratio of 100:1, using FuGENE® HD Transfection Reagent (Promega, E2311; Madison, WI, USA). After 48 hours, dual-luciferase activity was quantified using the Dual-Luciferase® Reporter Assay System (Vazyme, DD1205; Nanjing, China) according to the manufacturer’s protocol. Firefly luciferase activity was normalized to Renilla luciferase to control for transfection efficiency.

### 5-FU treatment and chemotherapy sensitivity assay

To evaluate chemotherapy response, HCT116 and RKO cells were treated with 0–160 µM 5-fluorouracil (5-FU; MedChemExpress, HY-90006, Monmouth Junction, NJ, USA) for 24 h. IC50 values were calculated using GraphPad Prism 8.0.2 (GraphPad Software, San Diego, CA, USA). Based on these values, a 10 µM concentration (approximately 1/4–1/2 of the IC50) was selected for subsequent cell viability assays.

For in vivo studies, 5-FU was freshly prepared and dissolved in PBS to a concentration of 4.5 mg/mL. Nude mice were treated with 5-FU at a dose of 23 mg/kg body weight via intraperitoneal injection, based on the recommended dosage from the manufacturer’s guidelines (MedChemExpress). The treatment was administered every 2 days for a total of 6 injections.

### In vivo tumorigenesis and metastasis assays

HCT116 cells (1 × 10^7^) treated according to the specific experimental conditions were then injected subcutaneously into 5-week-old male BALB/c nude mice (*n* = 5 per group; mice were randomly assigned to experimental groups using a random number table method; purchased from Guangdong Yaokang Biological Technology Co., Ltd., Guangdong, China). Tumor size and mouse weight were measured every 2-3 days. Tumor volume was calculated as (length × width^2^)/2. For 5-FU treatment, 7 days after subcutaneous injection, mice were treated with PBS (control) or 5-FU. Mice were sacrificed when the longest tumor diameter exceeded 1.5 cm, and tumors were photographed and weighed.

In addition, HCT116 cells (2 × 10^6^) with *PBX1* knockdown (shPBX1) or control (shNC) were injected via tail vein into 6 week-old male NOD-SCID mice (Vitonlihua Biotechnology Co., Ltd., China) to assess metastatic potential. Tumor metastasis was monitored by bioluminescent imaging starting from day 9 post-injection. Mice were injected with 150 μg D-luciferin (Solarbio, China, #D8390) per gram body weight and imaged 10 minutes after injection using the Perkin Elmer IVIS Lumina III vivo imaging system. Tumor growth and metastasis were quantified using Living Image software. The experiment was terminated when any mouse exhibited a weight loss greater than 5 g, in accordance with animal welfare guidelines.

Tumor measurements and outcome assessments were performed by investigators blinded to group allocation. All animal procedures were approved by the Institutional Animal Care and Use Committee of Shantou University Medical College (SUMC-2022-289).

### Statistical analysis

All statistical analyses were performed using IBM SPSS Statistics version 26.0 (IBM Corp., Armonk, NY, USA). Each experiment with statistical comparison was conducted with at least three independent biological replicates. Data are expressed as mean ± standard deviation (SD). Homogeneity of variance was tested using Levene’s test prior to t-test analysis. For comparisons with equal variance, standard Student’s t-test was applied; otherwise, Welch’s correction was used. One-way ANOVA followed by LSD post hoc test was used for multiple group comparisons. A P-value less than 0.05 was considered statistically significant. Detailed statistical methods and replicate numbers are provided in the corresponding figure legends.

## Supplementary information

Supplementary figure and tables

Original full length western blots for figures

checklist

## Data Availability

All raw sequencing data generated in this study have been deposited in the NCBI Sequence Read Archive (SRA). CUT&Tag datasets for PBX1 and H3K27ac are available under BioProject accession PRJNA1375645, with the associated BioSample accessions SAMN53690310–SAMN53690313. RNA-seq datasets from PBX1-knockdown and control HCT116 colorectal cancer cells are available under BioProject accession PRJNA1375649, with the associated BioSample accessions SAMN53690314–SAMN53690319.
