# Supplementary material for: Targeting the PBX1–BCL2L1 axis as a therapeutic strategy in colorectal cancer
Source: Cell Death Discov. 2026 May 5;12:280. doi: 10.1038/s41420-026-03139-2 (PMC13287496; doi:10.1038/s41420-026-03139-2)
Supplement: Supplementary file 1 — Supplementary figure and tables [file 41420_2026_3139_MOESM1_ESM.doc]

**Targeting the *PBX1–BCL2L1* Axis as a Therapeutic Strategy in Colorectal Cancer**

Hao Lin^1*^, Ting Su^2*^, Rulan Deng², Jie Li², Ying Liu², Xuanhao Lin³, Qiaoling Ke², Yijing Luo², Lele Meng², Bin Liang², Xuhong Song², Dongyang Huang²^#^, and Lingzhu Xie²^,4#^

¹ Department of Gastroenterology, Shantou Central Hospital, Shantou, 515041, China
² Department of Cell Biology and Genetics, Key Laboratory of Molecular Biology in High Cancer Incidence Coastal Chaoshan Area of Guangdong Higher Education Institutes, Shantou University Medical College, Shantou, 515041, China
³ Department of Biobank, Shantou Central Hospital, Shantou, 515041, China.

^4^ Department of Gastroenterology, First Affiliated Hospital of Shantou University Medical College, Shantou, 515041, China

^*^ These authors contributed equally to this work.

^#^Correspondence: Lingzhu Xie ( [lzxie@stu.edu.cn](mailto:lzxie@stu.edu.cn) ) or Dongyang Huang ( [huangdy@stu.edu.cn](mailto:huangdy@stu.edu.cn) )

# Supplementary figure

#
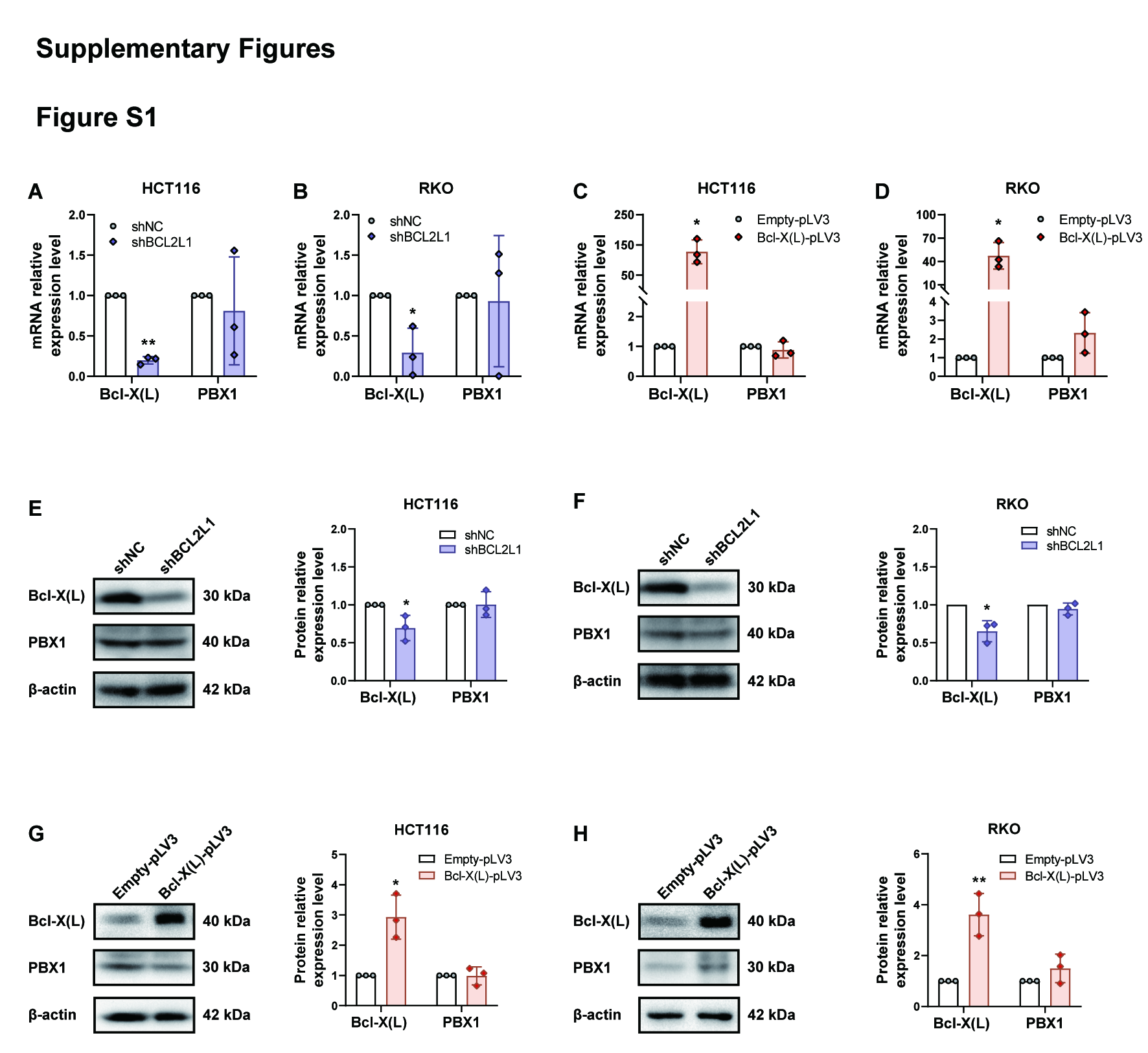


**Figure S1. BCL2L1 modulation does not significantly affect PBX1 expression or activity in CRC cells.** (A-D) qPCR analysis of PBX1 mRNA levels in HCT116 and RKO colorectal cancer cells following silencing or overexpression of BCL2L1. No significant changes in PBX1 mRNA levels were observed in either cell line. (E-H) Western blot analysis of PBX1 protein expression in HCT116 and RKO cells following modulation of BCL2L1. PBX1 protein levels remained unchanged upon silencing or overexpression of BCL2L1 in both cell lines. Significance for all data was determined by the independent samples t-test. Data are shown as mean ± S.D., n ≥ 3. **P* < 0.05, ***P* < 0.01.

# Supplementary tables

**Table S1.** Descriptive statistics of PBX1 expression distribution in colorectal cancer.

|  | **Min** | **Q1** | **Med** | **Q3** | **Max** | **Upper whisker** | **N** |
| --- | --- | --- | --- | --- | --- | --- | --- |
| Normal | 104 | 766 | 1025 | 1461 | 7786 | 2430 | 377 |
| Tumor | 7 | 405 | 641.5 | 954 | 6070 | 1768 | 1450 |
| Metastatic | 112 | 432.5 | 661 | 882.5 | 4722 | 1527 | 99 |

Note: Expression data were analyzed using the TNMplot online tool (https://www.tnmplot.com), based on data from the TCGA and GTEx cohorts. N, number of samples; Q1, first quartile; Q3, third quartile.

**Table S2.** Primer sequences for real-time RT- qPCR

| **Primer Names** | **Primer sequences** |
| --- | --- |
| PBX1-F | 5’- CAGTGGAGCATTCAGATTACAG -3’ |
| PBX1-R | 5’- TCGCAGGAGATTCATCACG -3’ |
| Bcl-xL-F | 5’- CGTGGAAAGCGTAGACAAGG-3’ |
| Bcl-xL-R | 5’- GGTGGTCATTCAGGTAAGTGG -3’ |
| Bcl-xS-F | 5’- GCTTTGAACAGGATACTTTTG -3’ |
| Bcl-xS-R | 5’- GGTAGAGTGGATGGTCAGTG -3’ |
| β-actin-F | 5’- TTGGCAATGAGCGGTTCC -3’ |
| β-actin-R | 5’- AGACAGCACTGTGTTGGC -3’ |

Note：The qPCR primers for Bcl-xL were designed to target a unique region specific to Bcl-xL (a segment absent in Bcl-xS).


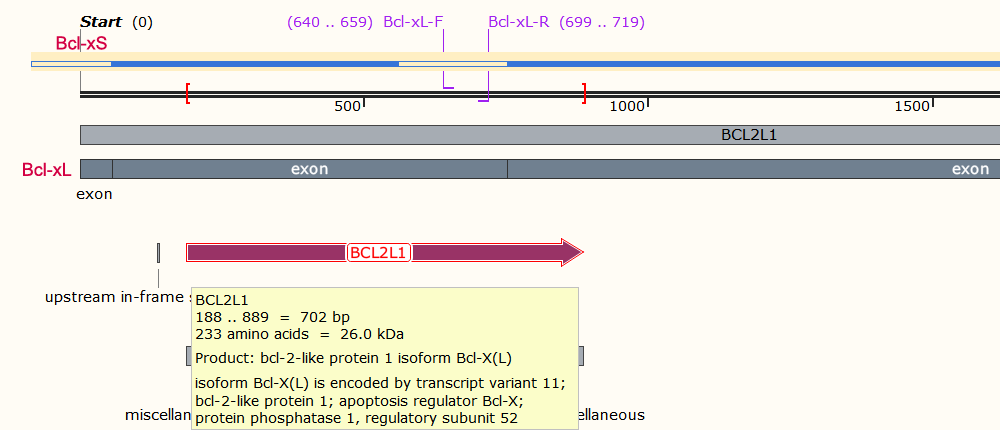


Note：The upstream primer for Bcl-xS was designed at the junction of exons 2 and 3, with a sequence unique to Bcl-xS.


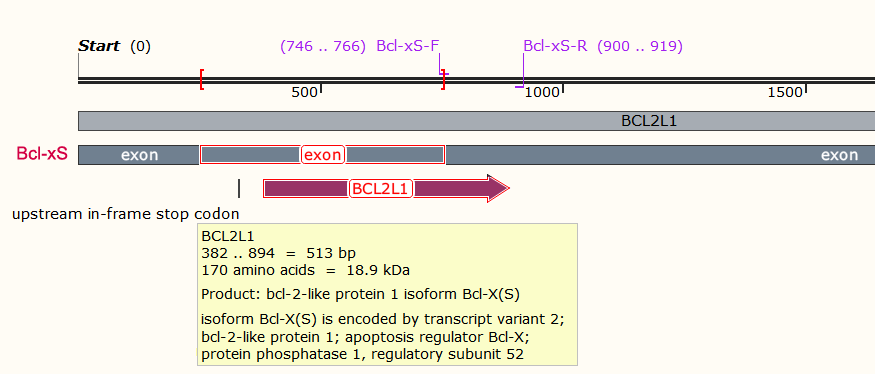


**Table S3.** Primer sequences for ChIP

| **Primer Names** | **Primer sequences** |
| --- | --- |
| Anti-PBX1-F | 5’-CCTACCTGGCTGACTGCTC-3’ |
| Anti-PBX1-R | 5’-CACTCCCTTTGCGTCTCG-3’ |
| Anti-H3K27ac-F | 5’-CTCTCCGCCTCCTACTGG-3’ |
| Anti-H3K27ac-R | 5’-GCCTTCAACATCACAGACAG-3’ |

**Table S4.** Primer sequences for dual-luciferase reporter assay

| **Primer Names** | **Primer sequences** |
| --- | --- |
| Promoter_homologous_F | 5’- atctgcgatctaagtaagcttGGCTGATTGACTGATGAGTGACTGAC -3’ |
| Promoter_homologous_R | 5’- cagtaccggaatgccaagcttTCTTTGTGGGTCTTACGAAGGTCTGG -3’ |
| Promoter_segment1_homologous_F | 5’- atctgcgatctaagtaagcttCCTCCCTGCGTCCCTCACTG-3’ |
| Promoter_segment1_homologous_R | 5’-cagtaccggaatgccaagctTTGGTTTCTTTGTGGGTCTTACGAAGG -3’ |
| Promoter_segment2_homologous_F | 5’- atctgcgatctaagtaagcttGTGCGTGACAGCCGTTGCG -3’ |
| Promoter_segment2_homologous_R | 5’- cagtaccggaatgccaagcttTCTCAATGGGGTTCAAGGTTTCAGTG -3’ |
| Promoter_segment3_homologous_F | 5’- atctgcgatctaagtaagcttAAACTGACGGACGGATGAAATAGGC -3’ |
| Promoter_segment3_homologous_R | 5’- aagcttggcattccggtactgGGAAGCGGGACGGCGAAGG -3’ |
