# Supplementary figures and images for: Targeting the PBX1–BCL2L1 axis as a therapeutic strategy in colorectal cancer
Source: Cell Death Discov. 2026 May 5;12:280. doi: 10.1038/s41420-026-03139-2 (PMC13287496; doi:10.1038/s41420-026-03139-2)

Figure 1

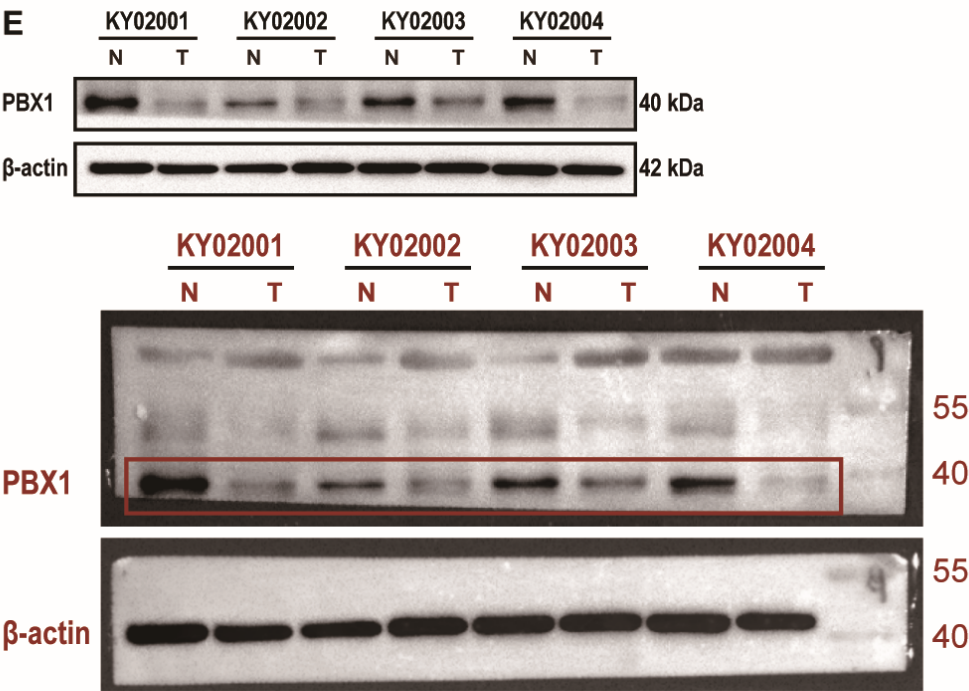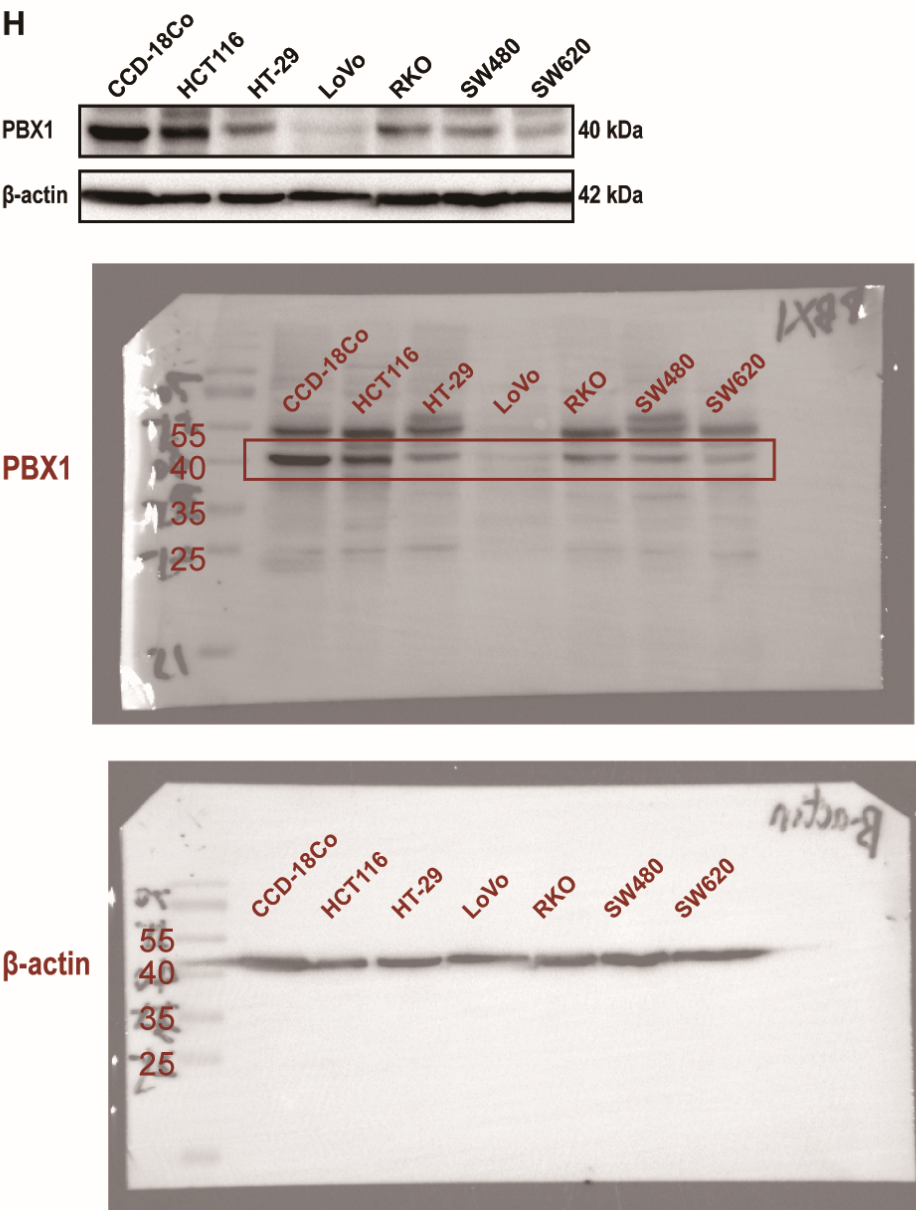

Figure 2

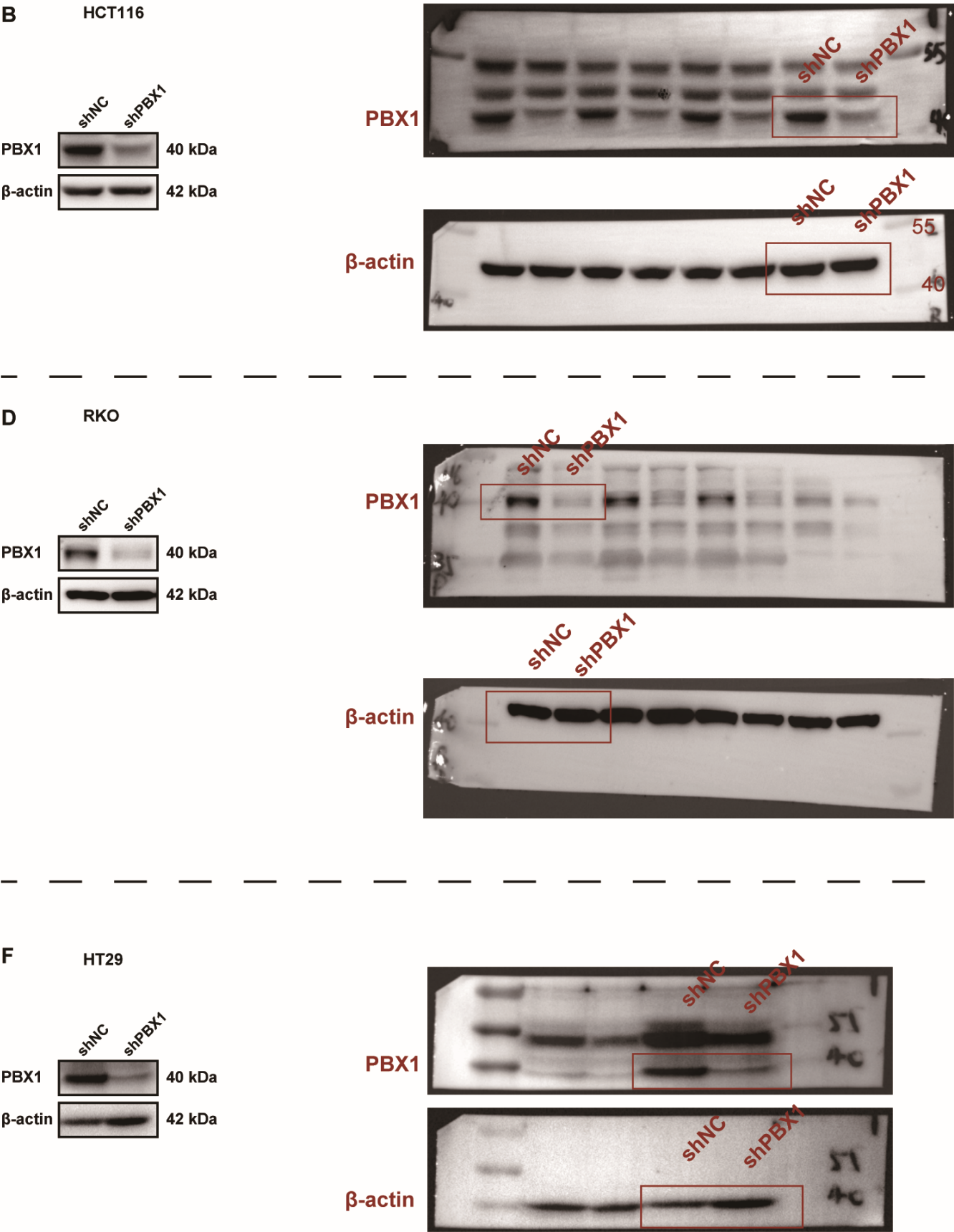

### Figure 3

**B** **HCT116**

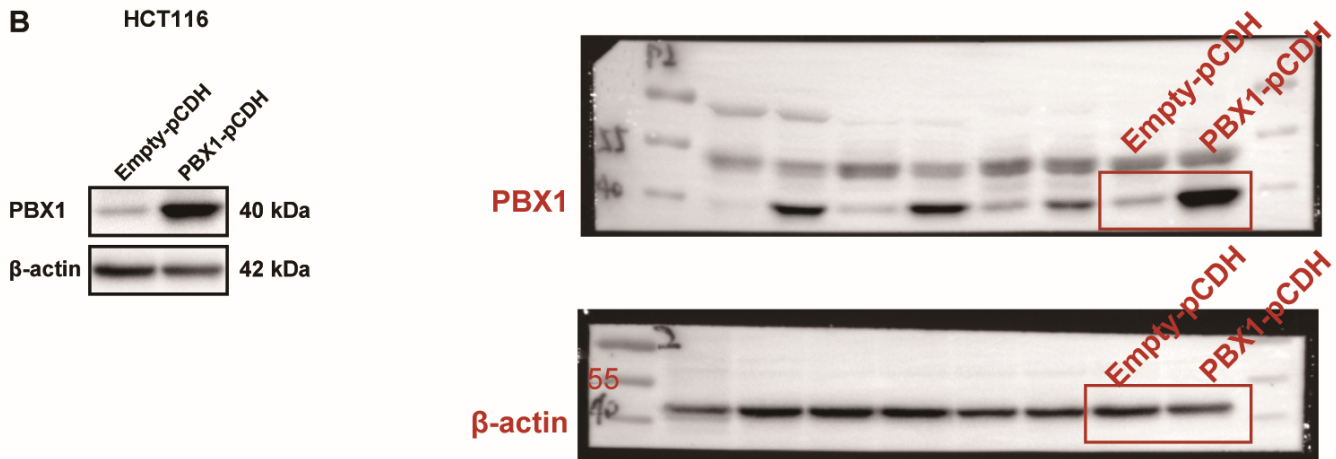

**D** **RKO**

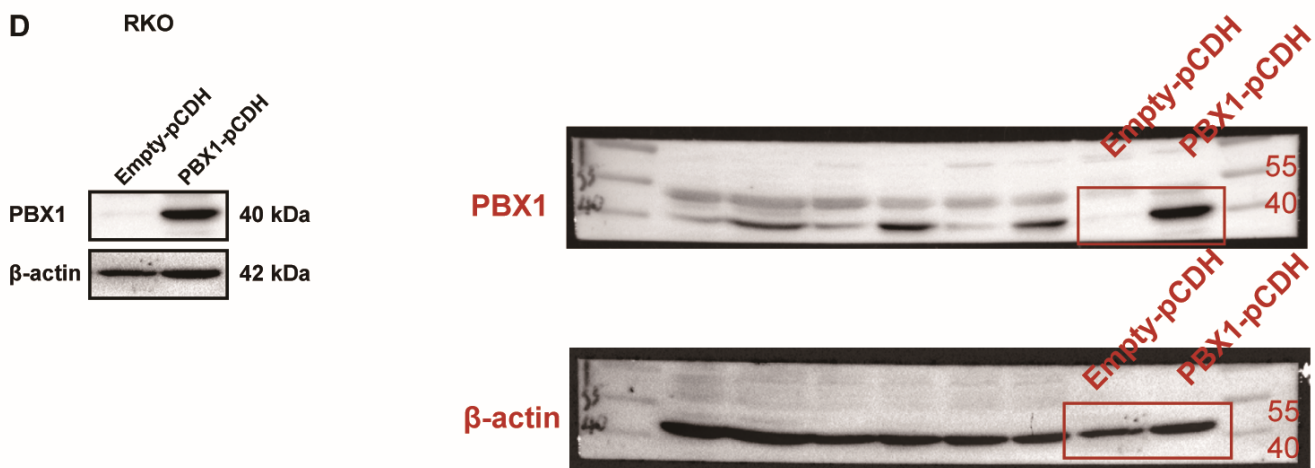

**F**      **LoVo**

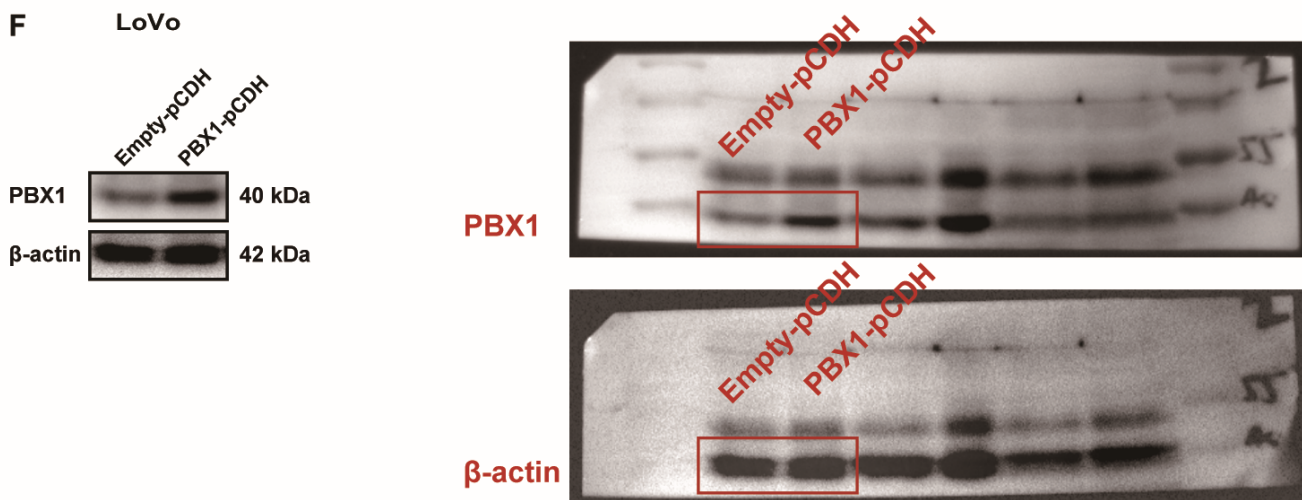

Figure 6

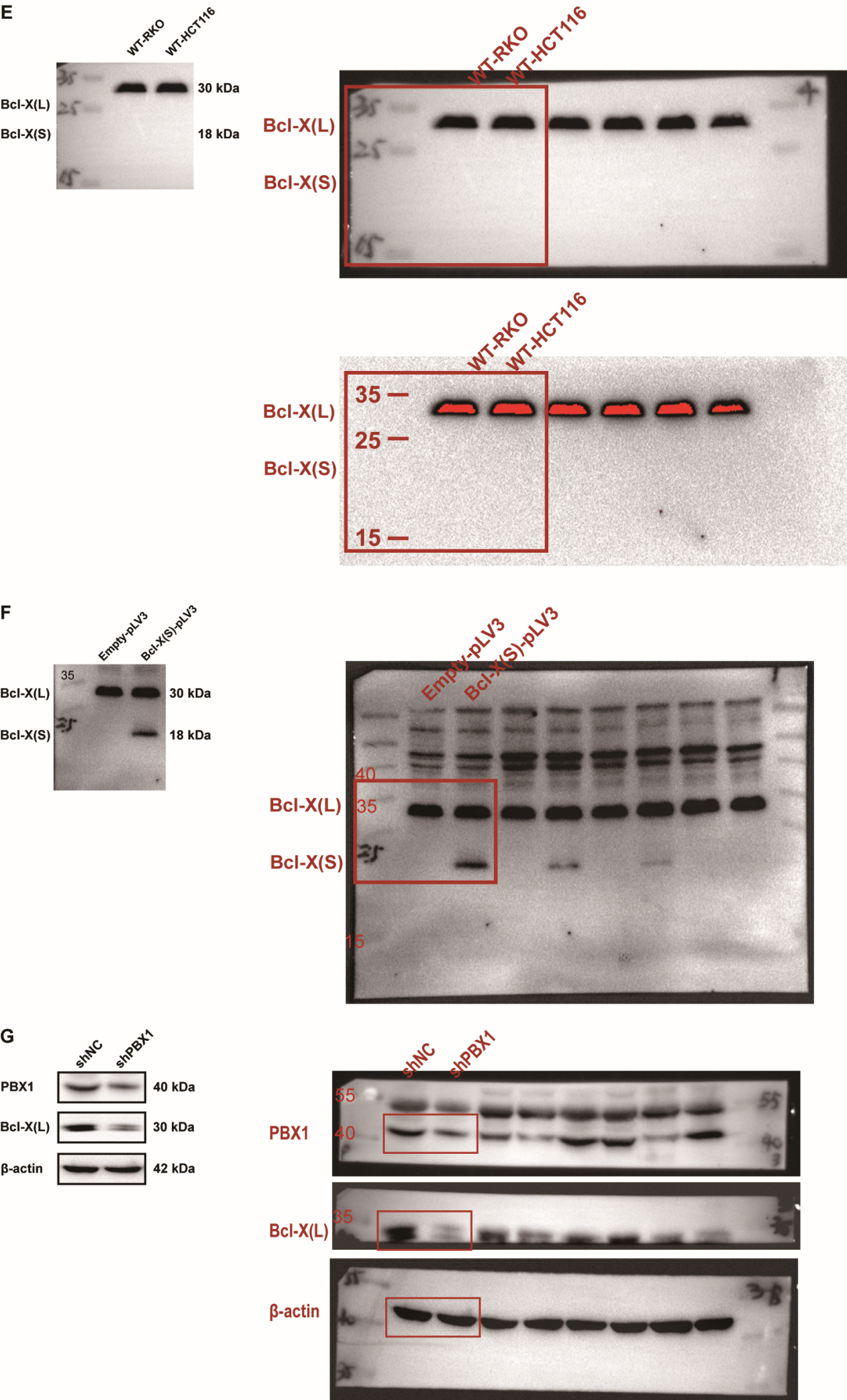

Figure 6

I

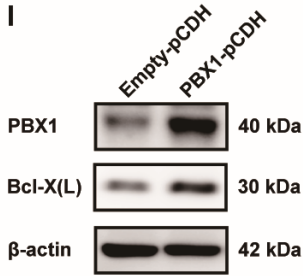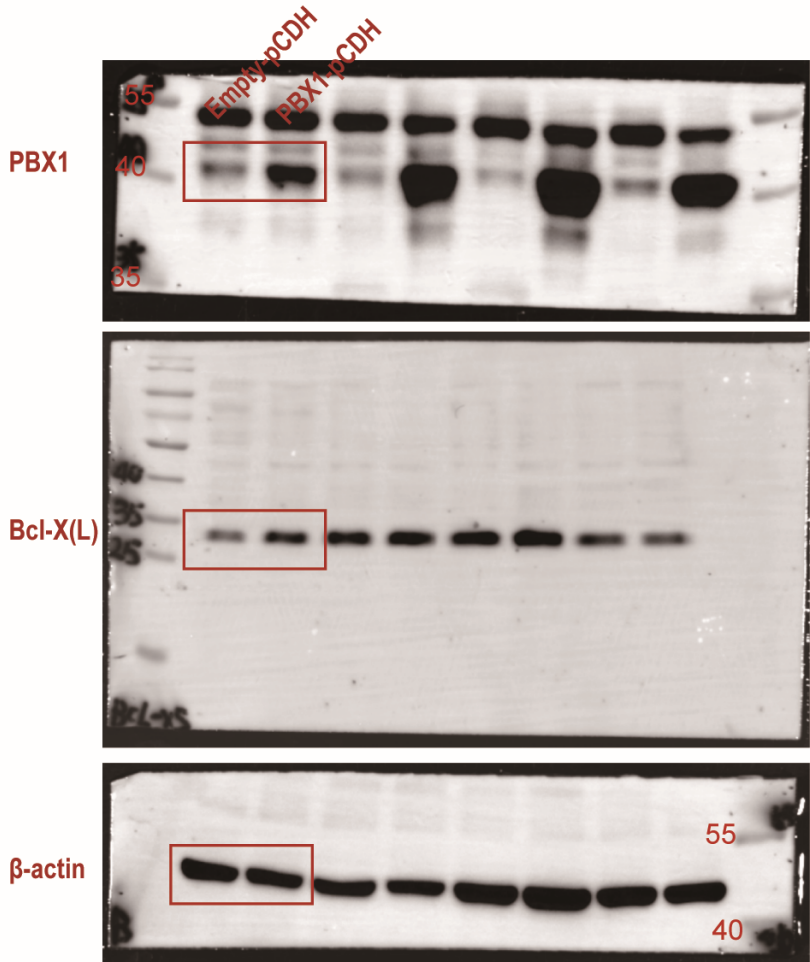

K

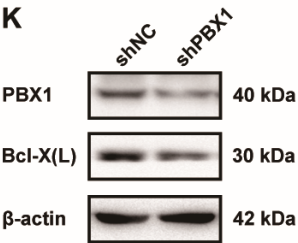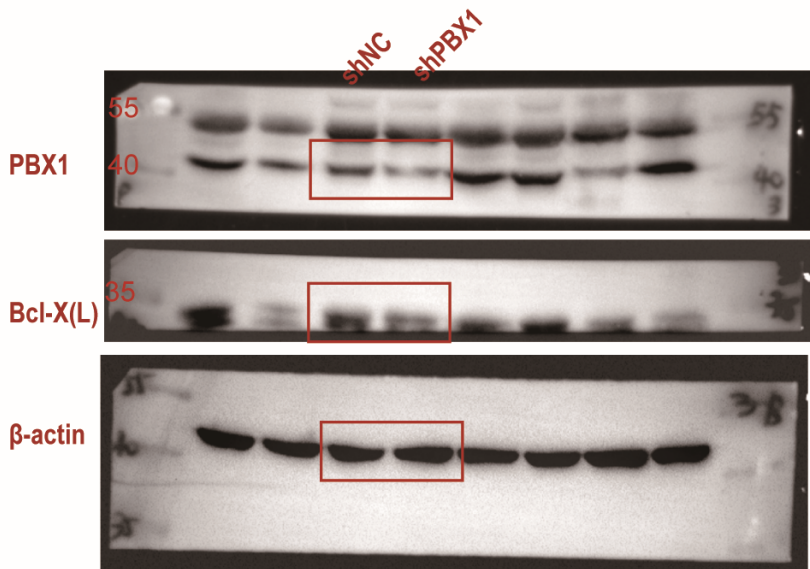

M

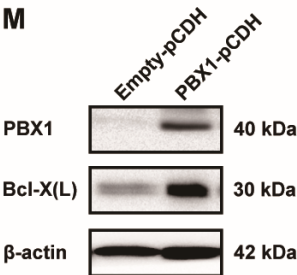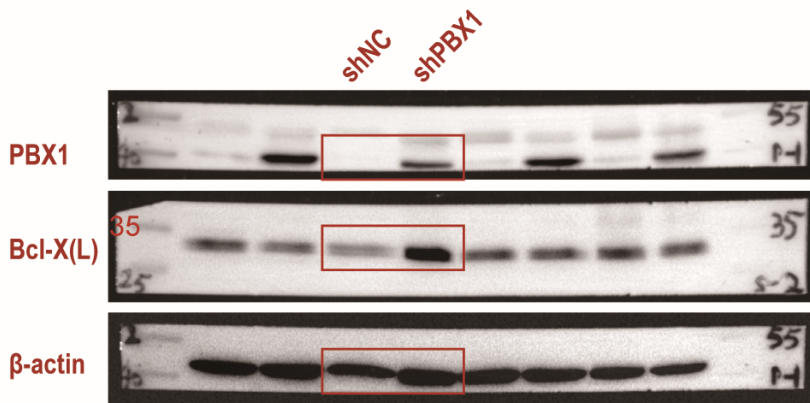

Figure 7

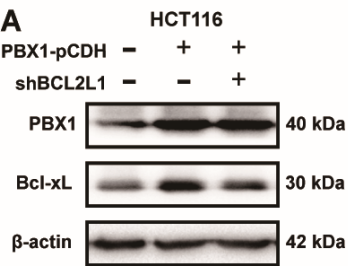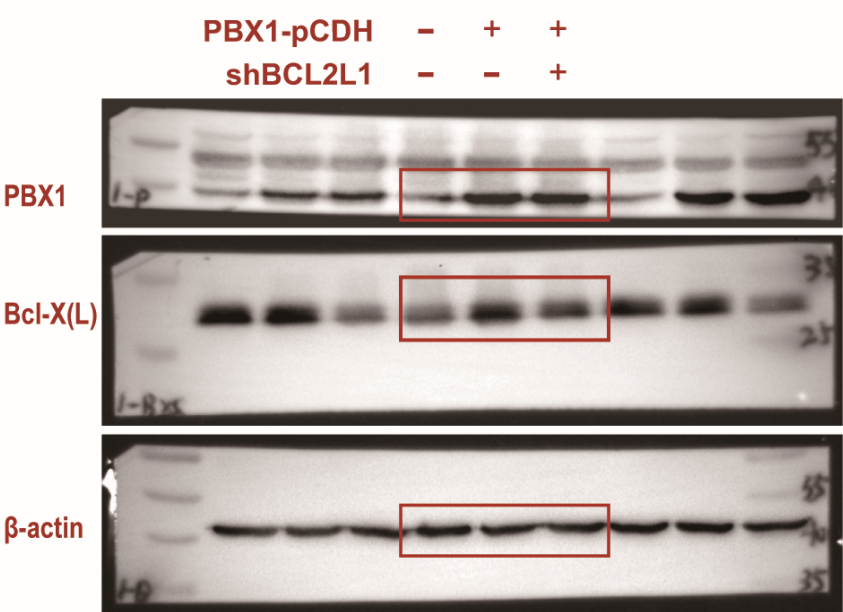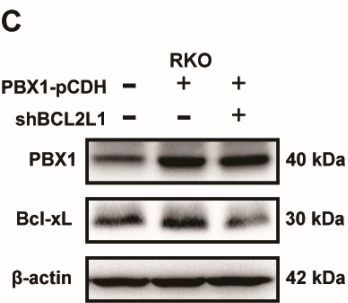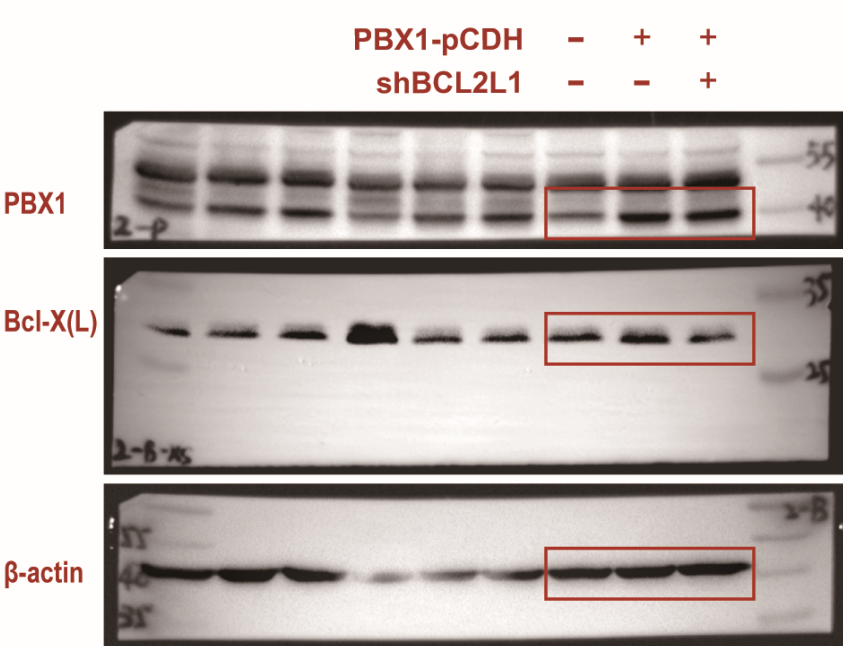

Supplementary Figures

Figure S1

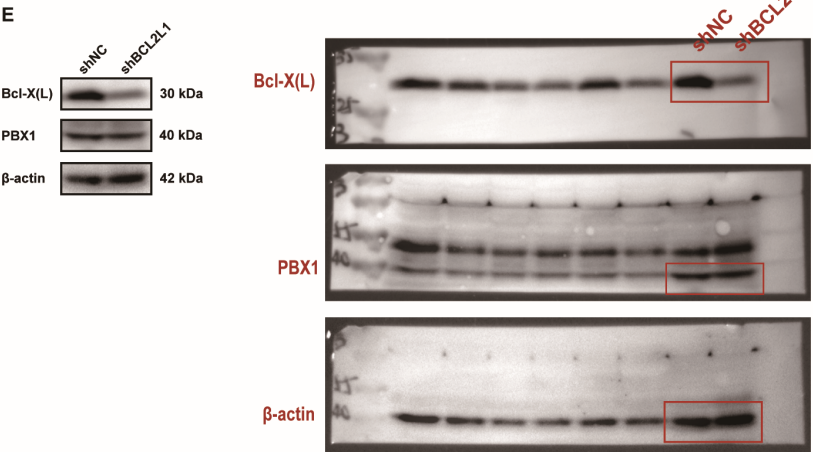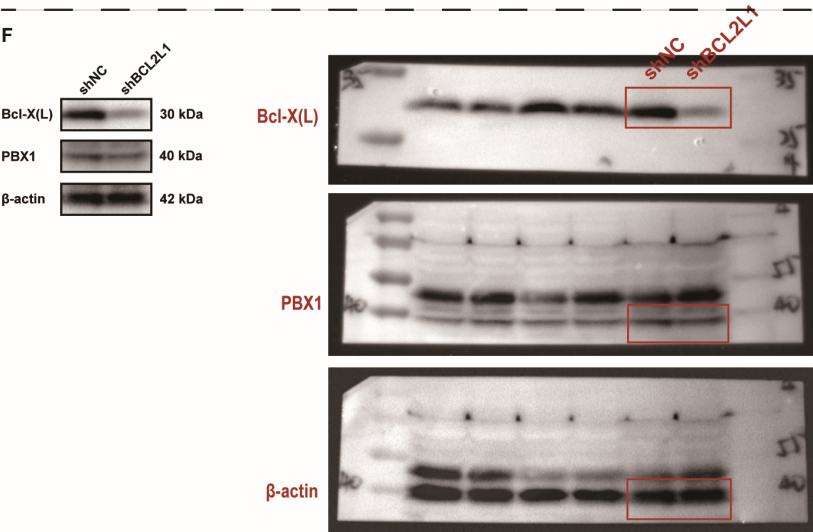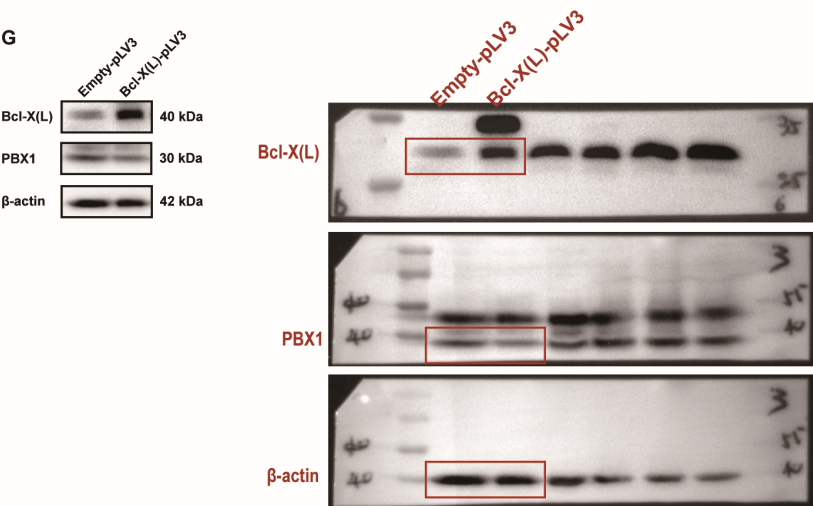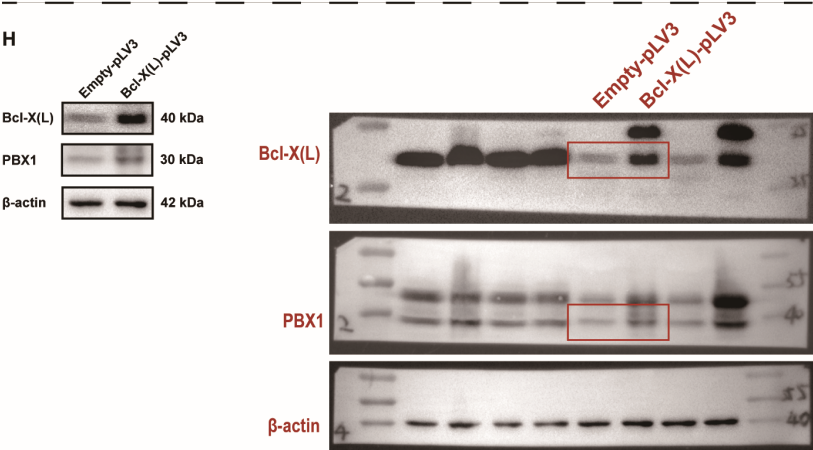

Supplement: Supplementary file 2 — Original full length western blots for figures [file 41420_2026_3139_MOESM2_ESM.pdf]
